# Insectivorous bats selectively source moths and eat mostly pest insects on dryland and irrigated cotton farms

**DOI:** 10.1002/ece3.5901

**Published:** 2019-12-12

**Authors:** Heidi Kolkert, Rose Andrew, Rhiannon Smith, Romina Rader, Nick Reid

**Affiliations:** ^1^ Ecosystem Management School of Environmental and Rural Science University of New England Armidale NSW Australia

**Keywords:** biocontrol, diet analysis, ecosystem services, integrated pest management, metabarcoding, natural pest control

## Abstract

Insectivorous bats are efficient predators of pest arthropods in agroecosystems. This pest control service has been estimated to be worth billions of dollars to agriculture globally. However, few studies have explicitly investigated the composition and abundance of dietary prey items consumed or assessed the ratio of pest and beneficial arthropods, making it difficult to evaluate the quality of the pest control service provided. In this study, we used metabarcoding to identify the prey items eaten by insectivorous bats over the cotton‐growing season in an intensive cropping region in northern New South Wales, Australia. We found that seven species of insectivorous bat (*n* = 58) consumed 728 prey species, 13 of which represented around 50% of total prey abundance consumed. Importantly, the identified prey items included major arthropod pests, comprising 65% of prey relative abundance and 13% of prey species recorded. Significant cotton pests such as *Helicoverpa punctigera* (Australian bollworm) and *Achyra affinitalis* (cotton webspinner) were detected in at least 76% of bat fecal samples, with *Teleogryllus oceanicus* (field crickets), *Helicoverpa armigera* (cotton bollworm), and *Crocidosema plebejana* (cotton tipworm) detected in 55% of bat fecal samples. Our results indicate that insectivorous bats are selective predators that exploit a narrow selection of preferred pest taxa and potentially play an important role in controlling lepidopteran pests on cotton farms. Our study provides crucial information for farmers to determine the service or disservice provided by insectivorous bats in relation to crops, for on‐farm decision making.

## Graphical abstract

Insectivorous bats consumed consistent proportions of Lepidoptera (frequency and abundance) over the cotton‐growing season, despite a pronounced temporal shift in the composition of available arthropod prey orders in cotton crops.

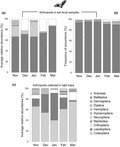

## 1 INTRODUCTION

Insectivorous bats consume a wide range of arthropods, some of which are considered major agricultural pests worldwide (Federico et al., 2008; Kunz, Braun de Torrez, Bauer, Lobova, & Fleming, 2011; Maine & Boyles, 2015; McCracken et al., 2012; Williams‐Guillen, Perfecto, & Vandermeer, 2008). Given that insectivorous bats consume 30%–100% of their body weight in prey each night (Kunz et al., 2011; Kunz, Whitaker, & Wadanoli, 1995; Kurta, Bell, Nagy, & Kunz, 1989), their potential to significantly increase agricultural productivity by suppressing pest arthropods is high. This pest control service has been estimated to be worth billions of dollars to agriculture globally by decreasing insect crop damage and increasing yield (Boyles, Cryan, McCracken, & Kunz, 2011; Cleveland et al., 2006; Maine & Boyles, 2015; Naylor & Ehrlich, 1997). However, few studies have explicitly investigated the composition and abundance of dietary prey items or assessed the ratio of pest and beneficial arthropods consumed, making it difficult to assess the quality of the pest control service provided by bats.

Many insectivorous bats are opportunistic predators (Heim et al., 2017) or selective opportunists choosing particular insect families from a variety of taxa available (McCracken et al., 2012; Murray & Kurta, 2002). Bats also actively search areas with abundant prey sources such as pest outbreaks in agricultural systems (Charbonnier, Barbaro, Theillout, & Jactel, 2014; Lee & McCracken, 2005; Müller et al., 2012; Ober & Hayes, 2008), indicating that insectivorous bats are able to adjust their predatory activity in relation to prey abundance (Gonsalves, Law, Webb, & Monamy, 2013b; Heim et al., 2017; Kennard, 2008; Lee & McCracken, 2005). For example, *Tadarida brasiliensis* (Brazilian free‐tailed bat) tracks and consumes large populations of *Helicoverpa zea* in cotton and corn crops in North America (Federico et al., 2008; Krauel, Ratcliffe, Westbrook, & McCracken, 2018; Lee & McCracken, 2002, 2005) and can deplete local pest insect populations within one growing season (Federico et al., 2008). However, little is known about the breadth of arthropods consumed by bats in agroecosystems, a critical step in understanding their “total” contribution to pest suppression.

Advances in molecular methods have facilitated the identification of cryptic dietary items and enabled predator–prey interactions to be revealed to a fine taxonomic level (Pompanon et al., 2012). Metabarcoding is one method that has been used to examine the impact of bats as control agents of selected arthropod pests (Bohmann et al., 2011; Brown, Braun de Torrez, & McCracken, 2015; Burgar et al., 2014; Krauel et al., 2018) and monitor fluctuations in insect pests through bat‐scat assays (Maslo et al., 2017). Metabarcoding can identify prey species that are overlooked via traditional microscopic dietary analysis methods, such as partially digested prey items (Pompanon et al., 2012). Qualitative and semiquantitative applications of metabarcoding can illustrate the efficiency of insectivorous bats as pest control agents as they enable identification of prey species and semiquantitative estimation of relative abundances (based on “relative read” abundance) in diets (Deagle et al., 2018; Thomas, Deagle, Eveson, Harsch, & Trites, 2016). Although semiquantitative estimates of relative abundance are not without bias (Pompanon et al., 2012; Thomas et al., 2016), they may provide a more accurate view of population‐level diet variation than traditional methods (Deagle et al., 2018).

Insectivorous bats exploit major lepidopteran cotton pests in the genus *Helicoverpa* (cotton bollworm or corn earworm moth) in agricultural systems worldwide (Brown et al., 2015; Krauel et al., 2018; Lee & McCracken, 2005; McCracken et al., 2012). However, the rise of transgenic cotton has significantly reduced the number of *Helicoverpa* and other lepidopteran pests in intensive cropping landscapes (Whitehouse, Wilson, & Fitt, 2005; Williams, Wilson, & Vogel, 2011). Transgenic cotton has also modified arthropod communities (Whitehouse et al., 2005; Zhao, Ho, & Azadi, 2011) and the arthropod prey available to insectivorous bats (Federico et al., 2008). Understanding which prey items insectivorous bats consume in transgenic cotton is a significant knowledge gap, given that transgenic cotton accounts for the majority of cotton grown globally, including 4.58 million ha in the United States alone (ISAAA, 2017). In Australia, 99.5% of cotton grown is transgenic. Cotton is an important agricultural export in Australia and integral to the social fabric and viability of cotton‐growing rural communities, contributing A$2 billion dollars annually (Cotton Australia, 2017). Thus, identification of prey to the lowest taxonomic level is vital to determine the extent to which insectivorous bats provide a pest control service or disservice to high‐value commodity crops such as transgenic cotton and corn.

This study investigated insectivorous bat diets to determine the range of prey items consumed and therefore evaluate the effectiveness of bats as pest regulators. Insectivorous bats are assumed to provide a pest regulation service, yet major knowledge gaps remain in relation to the diversity of insectivorous bats present, and information on what they eat in transgenic cotton crops. We asked three questions: (a) How diverse is the insectivorous bat community foraging in cotton crops over the cotton‐growing season (November–March)? (b) Which prey items are consumed by insectivorous bats, and what are the relative quantities of pest and beneficial arthropods consumed? (c) Do the arthropods in insectivorous bat diets reflect arthropod prey abundance across the summer‐growing season? This study enabled species‐level dietary exploration of an insectivorous bat community to evaluate the contribution to natural pest control in transgenic cotton‐growing landscapes.

## 2 MATERIALS AND METHODS

### 2.1 Study sites and sample collection

**Figure 1:**
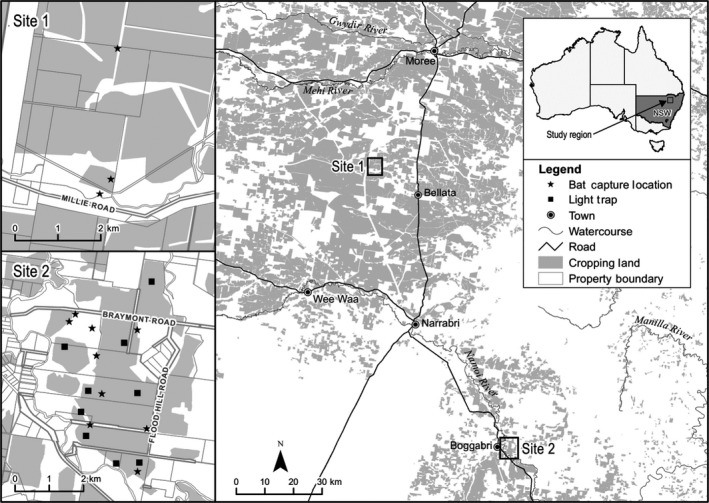
Location of study region

Research was undertaken during the 2013–14 and 2014–15 summer cotton‐growing seasons on two cotton farms in the Namoi and Gwydir Valleys in northern New South Wales (NSW), Australia (Figure 1): Site 1 was a dryland cotton farm near Bellata (29°49′28.7″S 149°39′10.8″E), and Site 2 was an irrigated cotton farm near Boggabri (30°43′15.4″S 150°04′52.5″E). These farms were located in the “humid subtropical” Köppen‐Geiger climate zone (Kottek, Grieser, Beck, Rudolf, & Rubel, 2006) in the Brigalow Belt South Bioregion (Environment Australia, 2000). Bollgard II^®^ cotton (Bt‐cotton), containing two genes derived from the common soil bacterium, *Bacillus thuringiensis*, was grown on both farms, with 10% of the cropping area dedicated to unsprayed cotton refuge crops (conventional cotton and pigeon pea).

Trapping for insectivorous bats occurred over a total of 48 nights, including eight nights in December 2014 at Site 1 and eight nights each month during the cotton‐growing season (November 2015–March 2016) at Site 2. Bats were captured using mist nets (Ecotone) and harp traps (Faunatech) placed in cotton crops or on the edge of crops (Figure 1). Mist nets and harp traps were moved to a different sampling location (Figure 1) each night at each site (Marques et al., 2013). On each occasion, we used three mist nets (12 × 2.5 m, 15 × 2.5 m, and 18 × 2.5 m; 5 shelves, Denier 110, 16 mm mesh size) and three harp traps (standard 4.2‐m^2^ two‐bank traps) that were opened at dusk and closed at midnight. All captured bats were placed individually in clean calico bags and transferred to a holding area until the following dusk. Bat identification was undertaken using Churchill (2009) and corroborated by echolocation recording on release of each individual. Fecal pellets from each bat (2–8 pellets) were collected from the bag with sterilized tweezers. Each fecal sample was stored in a labeled sterile 1.5‐ml microcentrifuge tube and frozen (−4°C) within 2 hr. Samples were then transferred to a −20°C freezer until DNA extraction. Fecal samples left in traps by escaped bats were noted as “unknown” species.

Invertebrate light traps were set in the center of cotton crops to estimate prey abundance in parallel with bat surveys at Site 2 (Figure 1). The custom‐made light traps were specially designed to be positioned above a growing crop (Peter Gregg, personal communication, 2016). A large fiberglass cone (750 mm diameter × 500 mm high, smooth on the inside and sourced from Fiberglass Moulding Pty Ltd) was placed inside a 60‐L garbage bin, with a strip of 12‐V ultraviolet LED lights taped to an aluminum ring resting inside the cone to attract flying invertebrates. The LEDs were connected to a 12‐V battery and 10‐W solar panel (SGM‐10W, Solar Australia). Traps were emptied each morning. Invertebrate collections were immediately transferred into 70% ethanol and dried in the laboratory at 40°C prior to identification to arthropod order (>1 mm) and weighing. Invertebrates <1 mm were removed from further analysis. The percentage of each invertebrate order was then pooled with other light trap samples from the same night and farm. Given that most insectivorous bat species feed on night‐flying insects, light trapping is commonly used to measure insect abundance and understand bat–prey dynamics (Froidevaux, Fialas, & Jones, 2018; Gonsalves, Law, et al., 2013b; Krauel et al., 2018; McCracken et al., 2012). To avoid the attractiveness (or avoidance) of bats to light traps, they were placed at least 250 m from where bats were captured (Froidevaux et al., 2018).

### 2.2 DNA extraction, sequencing, and metabarcoding

A 157‐bp section of the cytochrome c oxidase subunit 1 (COI) mitochondrial DNA barcoding region was amplified using arthropod‐specific primers ZBJ‐ArtF1c and ZBJ‐ArtR2c (Table 1) developed to analyze the diet of insectivorous bats (Zeale, Butlin, Barker, Lees, & Jones, 2011). The COI region provides high taxonomic resolution and is ideal for identifying species and intraspecific variation (Krehenwinkel et al., 2017; Rubbmark, Sint, Horngacher, & Traugott, 2018). The Zeale et al. (2011) primers have been evaluated and used successfully in several dietary studies across a wide range of arthropod orders (Alberdi, Aizpurua, Gilbert, Bohmann, & Mahon, 2018; Bohmann et al., 2011; Razgour et al., 2011; Zeale et al., 2011).

**Table 1:** Primer sequencing details

Target	ZBJ‐ArtF1c–ZBJ‐ArtR2c
ZBJ‐ArtF1c	AGATATTGGAACWTTATATTTTATTTTTGG
ZBJ‐ArtR2c	WACTAATCAATTWCCAAATCCTCC
Application	Amplicon sequencing
Read Length	300bpPE

DNA extraction and PCR amplification were carried out by the Melbourne Node of the Australian Genome Research Facility (AGRF). DNA was extracted from fecal samples using The PowerLyzer^®^ PowerSoil^®^ DNA Isolation Kit (MO BIO, #12855) following the manufacturer's instructions. This included a bead beating (homogenization) step and column‐based purification. PCR was completed by the AGRF Brisbane Node using the Illumina Nextera XT Index Kit (Illumina, USA #FC‐131), 2‐stage PCR design. PCR amplicons were generated using the primers shown in Table 1, with AmpliTaq Gold^®^ 360 Master Mix (Life Technologies, #4398881) for the primary PCR. A secondary 8‐cycle PCR to index the amplicons was performed with TaKaRa Taq™ DNA Polymerase (Clontech, USA #R001B). PCR thermal cycling conditions were as follows: 95°C for 5 min followed by 40 cycles of 95°C for 15 s; an annealing temperature of 52°C for 30 s and 72°C for 30 s; and final extension at 72°C for 7 min. The concentration of the resulting amplicons was quantified by fluorometry (Invitrogen Picogreen) and normalized. The equimolar pool was then measured by qPCR (KAPA, Roche) and visualized on the Bioanalyser (Agilent).

Sequencing was completed by the AGRF Melbourne Node on the Illumina MiSeq with 2 × 150‐bp paired‐end v2 chemistry. Image analysis was performed in real time by the MiSeq Control Software (MCS, version 2.5.0.5) and Real Time Analysis (RTA, version 1.18.54). The Illumina pipeline (bcl2fastq, version 2.17.1.14) was then used to process the sequence data. Paired‐end reads were combined by aligning the forward and reverse reads using PEAR (version 0.9.5; Zhang, Kobert, Flouri, & Stamatakis, 2014). Primers and adapters were trimmed using Seqtk (version 1.0; Li, 2012). Trimmed sequences were processed using Quantitative Insights into Microbial Ecology (QIIME version 1.8; Caporaso et al., 2010), USEARCH (version 8.0.1623; Edgar, 2010; Edgar, Haas, Clemente, Quince, & Knight, 2011), and UPARSE (Edgar, 2013) software. Using USEARCH tools (Edgar, 2010), reads were quality‐filtered, full‐length duplicate sequences were removed, and sequences were sorted by abundance. Singletons (unique reads) were discarded. The trimmed, quality‐filtered sequences were clustered into prey OTUs with a 97% similarity threshold. The number of reads in each OTU and the estimated relative proportion were recorded for downstream analysis of dietary diversity.

The taxonomy of OTUs was assigned using the NCBI database nucleotide Basic Local Alignment Search Tool (Altschul, Gish, Miller, Myers, & Lipman, 1990). OTU sequences were assigned to reference sequences at species level with a minimum identity threshold of 97%, reflecting natural intraspecific divergence (Alberdi et al., 2018; Elbrecht & Leese, 2017), and an e‐value < 1^e−20^. There is no general rule as to dealing with identity thresholds, read errors, or discarding sequences (Pompanon et al., 2012). In this study, an OTU was classified as “unknown” when a taxonomic assignment fell below the similarity threshold or when a match was not found in the NCBI database (see Appendix S1 for further details). Those unable to be identified to species level were identified to order. Based on the taxonomic identification of OTUs, the relative abundance of sequences assigned to order and species level was used as a proxy to semiquantify the relative abundance and richness of prey species in each sample (Deagle et al., 2018). Each taxonomically assigned prey species was then allocated to one of six categories (“pest,” “beneficial,” “pest likely,” “beneficial likely,” “unknown,” or “neutral”) based on literature detailing their impact in natural and agricultural systems, and in cotton specifically (Appendix S2).

### 2.3 Statistical analysis

All statistical analyses, diversity indices, and species accumulation curves were performed using PRIMER v7 software (Clarke & Gorley, 2015) with the PERMANOVA+ add‐on (v.1.0.8; PRIMER‐E, Plymouth Marine Laboratory, UK) for the analysis of nonparametric multivariate or univariate ecological datasets (Anderson, 2001). Two relative abundance datasets were used to analyze dietary composition: (a) the OTU dataset (Appendix S3) and (b) the taxonomically assigned prey species dataset (728 unique species). Dietary richness was initially explored by examining the differences in the frequency of OTUs and taxonomically assigned prey species between bat fecal samples. OTU relative abundance was calculated using the proportion of total reads in a sample (determined by sequencing). The richness and relative abundance of each prey taxon was calculated using the OTUs taxonomically assigned to species level (728 unique species) and expressed as a percentage of identified prey species per bat sample.

To ensure that estimates of total species and subsequent species diversity calculations were reliable, species accumulation curves were constructed for the number of species and the number of OTUs in bat fecal samples, randomizing the samples 9,999 times. Four nonparametric richness estimators were used: Chao2, Bootstrap, Jackknife 1, and Jackknife 2. These emphasize the incidence of rare species, an accepted approach for estimating species richness in ecological studies (Gotelli & Colwell, 2011; Cardoso, Rigal, Borges, & Carvalho, 2014). A K‐dominance curve (cumulative relative abundance against the log species rank) was constructed measuring abundance trends and inventory diversity using both datasets (results are shown in Appendix S4).

Permutational analysis of variance (PERMANOVA) was used to test for differences in richness and relative abundance of OTUs, arthropod orders, and prey species on Bray–Curtis matrices. PERMANOVA is a distance‐based nonparametric test with Pseudo‐*F* and *p*‐values obtained using permutation techniques (Anderson, 2001). PERMANOVA is recommended for examining complex community composition datasets with small sample sizes (Anderson, 2001; McArdle & Anderson, 2001). Unrestricted permutations of raw data were completed using 9,999 permutations. PERMANOVAs were conducted to assess the temporal variability in dietary composition among months of the cotton‐growing season at Site 2 (*n* = 5: November–March), and the spatial variability between farms at Site 1 (December 2014) and Site 2 (December 2015; see results in Appendix S5). A two‐way PERMANOVA was conducted with bat sex (two groups: male and female) and bat species with ≥ 6 samples (three groups: *Vespadelus vulturnus*, *Nytophilus geoffroyi*, and *Chalinolobus gouldi*) as factors. PERMANOVAs were also conducted on the prey species dataset with pest status of natural and modified systems (six groups: “pest,” “beneficial,” “pest likely,” “beneficial likely,” “unknown,” or “neutral”) and cotton pest status (six groups: as before) as factors. Following PERMANOVAs, PERMDISP was used to examine the homogeneity of dispersion (Anderson, 2006), based on mean distance to group centroid for all groups within each factor (9,999 permutations). Univariate analysis was undertaken for important single‐factor comparisons using a Euclidean distance matrix (Anderson). Variability in composition between months (*β* diversity) was measured using PERMDISP on a Bray–Curtis similarity matrix (presence/absence data), which is equivalent to the Sørensen index (Anderson, 2006; Anderson, Gorley, & Clarke, 2008).

To examine how representative consumed prey species were in relation to the prey available in the cotton landscape, average taxonomic distinctness (AvTD) and variation in taxonomic distinctness (VarTD) within each bat fecal sample were examined. AvTD and VarTD measure the average taxonomic breadth and evenness of a sample between every pair of species and the mean value recorded in the species dataset under random sampling (Clarke & Warwick, 1999, 2001; Ellingsen, Clarke, Somerfield, & Warwick, 2005). AvTD (Delta+) and VarTD (Lambda+) were calculated from the prey species dataset (728 species) and compared to 95% probability limits under random sampling using the TAXDTEST function, based on the Linnaean relatedness (see Appendix S1 for further information).

## 3 RESULTS

### 3.1 Bat species richness

In total, 58 individual insectivorous bats of seven species (all Vespertilionidae) were captured at the two sites (Table 2). Of these, 12 were male, 12 female, and the remainder of unknown sex (due to not being sexed or escaping traps prior to sexing). Two species, *Chalinolobus picatus* and *Vespadelus baverstocki*, are endangered in NSW, yet are relatively common in inland and arid areas. The diversity of insectivorous bats known to forage over cotton crops is considerably higher than those species captured in this study (approximately 20 species) and highlights the difficulty of trapping echolocating bats in open areas where they may detect and avoid traps.

**Table 2:** Details of scats collected from captured insectivorous bats

Species	Female	Male	Unknown^a^	Total
Site 1	–	–	13	13
*Chalinolobus picatus*	–	–	1	1
*Nyctophilus geoffroyi*	–	–	7	7
*Vespadelus baverstocki*	–	–	1	1
*Vespadelus vulturnus*	–	–	3	3
Unknown^a^	–	–	1	1
Site 2	12	12	21	45
*Chalinolobus gouldii*	1	4	1	6
*Chalinolobus morio*	–	1	–	1
*Nytophilus geoffroyi*	4	3	–	7
*N. geoffroyi* or *C. gouldii*	–	–	1	1
*Scotorepens greyii*	1	1	–	2
*V. vulturnus*	6	3	7	16
*V. vulturnus* or *N. geoffroyi*	–	–	1	1
Unknown	–	–	11	11
Total	12	12	34	58

a Unknown “sex” or “species” indicate that the bat was not sexed or escaped from a harp trap prior to identification or sexing. Two species (i.e., *N. geoffroyi* or *C. gouldii* and *V. vulturnus* or *N. geoffroyi*) indicate that a scat was collected from a trap that could have come from one of either species.

### 3.2 Sequencing and OTU assignment

Sequencing yielded 5,519,504 query reads, of which 5,412,769 (92%) passed quality filtering and were clustered into 2,760 OTUs (based on >97% identity). Of these, 1,154 OTUs (42%) were matched to species level based on BLASTn alignments in the reference database. Of the species matches, 196 OTUs had 100% similarity to the reference database. The 1,154 species matches were equivalent to 728 unique prey species (Table 3). The 728 species represented 72% of the relative abundance in the OTU dataset (Table 3), with the remaining 28% not meeting the species identity threshold.

**Table 3:** Contribution of top 13 prey species (>50% relative abundance of diet based on OTU read counts for each taxa) detected in fecal samples (based on 97% similarity and e‐value ≥ 1–20 with NCIB database, BOLD sequences)

Scientific name	Order	Pest status: Natural and modified systems	Type of impact to natural and modified systems	Known pest status: Cotton	Average relative abundance (%)	Maximum relative abundance (%)	Frequency of occurrence in bat fecal samples (%)
*Tenebrio molitor*	Coleoptera	Pest	Stored grain	Neutral	12.10	98.2	79.31
*Athetis tenuis*	Lepidoptera	Pest	Crop	Neutral	4.79	78.1	84.48
*Teleogryllus oceanicus*	Orthoptera	Pest	Crop	Pest	4.56	86.0	65.52
*Achyra affinitalis*	Lepidoptera	Pest	Crop	Pest	4.47	83.4	75.86
*Faveria tritalis*	Lepidoptera	Pest	Turf	Neutral	4.26	97.4	87.93
*Endotricha puncticostalis*	Lepidoptera	Pest	Crop	Neutral	4.26	86.2	93.10
*Olbonoma* sp. *ANIC21*	Lepidoptera	Neutral	Neutral	Neutral	4.10	84.3	74.14
*Armactica conchidia*	Lepidoptera	Pest likely	Emergent	Neutral	3.55	95.6	56.90
*Sceliodes cordalis*	Lepidoptera	Pest likely	Crop	Neutral	2.09	89.6	44.83
*Cyana* sp. *BOLD:AAN5000*	Lepidoptera	Pest likely	Crop	Neutral	1.90	33.1	62.07
*Ectopatria horologa*	Lepidoptera	Neutral	Neutral	Neutral	1.78	99.1	65.52
*Leucania stenographa*	Lepidoptera	Pest	Crop	Neutral	1.73	85.8	44.83
*Tathorhynchus fallax*	Lepidoptera	Pest	Emergent	Neutral	1.65	42.0	62.07

Note

Sequenced DNA read counts were used as a proxy to semiquantify abundance.

### 3.3 OTU richness

The species accumulation curves showed a similar pattern across all richness estimators, with the observed number of prey items detected close to saturation (see Appendix S4). All estimators reached 100% observed prey richness (asymptote) at 42 samples. Our study thus provided a reliable basis for assessing total prey species richness.

### 3.4 Prey composition

**Figure 2:**
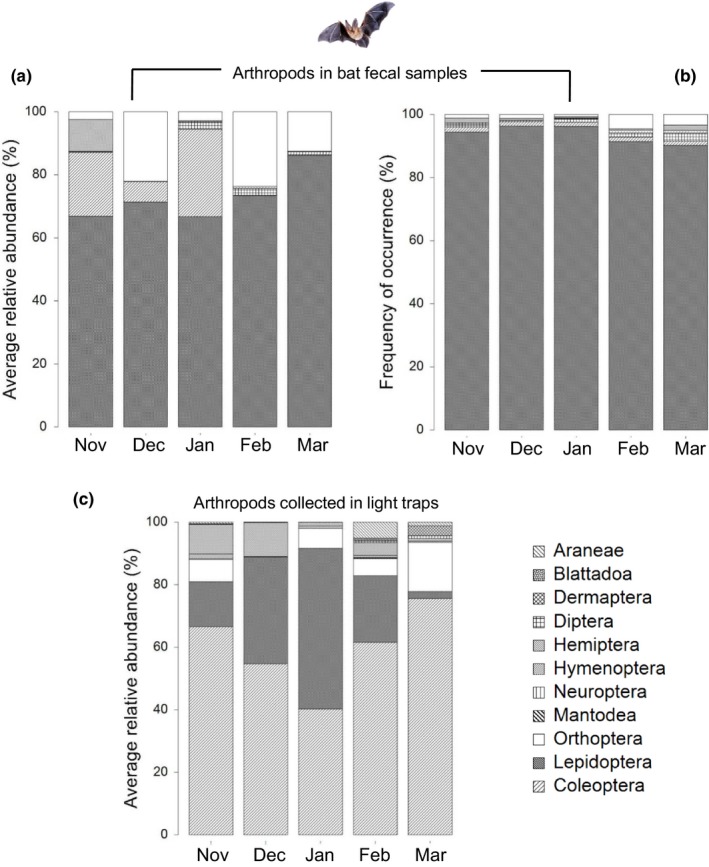
Comparison between the (a) relative abundance and (b) frequency of prey orders consumed by insectivorous bats at Site 2 (using DNA read counts as a proxy for abundance), and (c) prey available from light trap collections over the cotton‐growing season at Site 2. Based on >97% similarity and e‐value ≥ 1–20 with NCIB database, BOLD sequences, (c) shows the relative abundance of arthropod orders in light trap collections by month, *n* = 64 (November, January, and February, *n* = 11; December, *n* = 13; March, *n* = 18)

Thirteen species contributed >50% of the relative abundance of the prey species dataset (Table 3). The K‐dominance plot confirmed that few species contributed most of the relative abundance of items in each bat sample (see Appendix S4). Arthropod orders with the greatest relative abundance represented in the prey species dataset included Lepidoptera (75.2%), Coleoptera (14.0%), Orthoptera (6.6%), Diptera (2.3%), and Hemiptera (1.9%) (Table 4). The volume of Lepidoptera in bat diets was dominant throughout the cotton‐growing season despite the abundance of other potential prey taxa (Figure 2).

**Table 4:** Relative species abundance (%) and species richness (%) of pest and beneficial arthropod orders detected in bat fecal samples

Arthropod Order	Col	Dip	Hem	Hym	Lep	Neu	Ort	Tri	Total
Relative abundance									
Crop	0.94	0.54	0.22	–	36.39	–	6.57	–	44.67
Disease vector	–	0.67	–	–	0.00	–	–	–	0.67
Emergent	–	–	1.47	–	9.45	–	–	–	10.92
Forestry	–	0.00	–	–	1.41	–	–	–	1.41
None	0.09	0.10	0.21	0.00	21.08	–	–	0.04	21.53
Orchard	–	–	–	–	0.10	–	–	–	0.10
Parasitic	–	0.95	–	–	0.00	–	–	–	0.95
Predator	0.03	–	–	–	–	0.04	–	–	0.07
Soil	0.00	–	–	–	–	–	–	–	0.00
Stored grain	12.94	–	–	–	–	–	–	–	12.94
Turf	–	–	–	–	5.19	–	–	–	5.19
Unknown	–	–	–	–	1.51	–	–	–	1.51
Weed control	–	–	–	–	0.04	–	–	–	0.04
Total	14.00	2.26	1.89	0.00	75.17	0.04	6.57	0.04	100.00
Richness									
Crop	0.27	1.24	0.27	–	9.20	–	0.82	–	11.81
Disease vector	–	0.82	–	–	0.14	–	–	–	0.96
Emergent	–	–	0.27	–	2.06	–	–	–	2.34
Forestry	–	0.14	–	–	0.69	–	–	–	0.82
None	0.41	0.69	0.41	0.14	68.54	–	–	0.41	70.60
Orchard	–	–	–	–	3.02	–	–	–	3.02
Parasitic	–	0.41	–	–	0.14	–	–	–	0.55
Predator	0.27	–	–	–	–	0.41	–	–	0.69
Soil	0.14	–	–	–	–	–	–	–	0.14
Stored grain	0.41	–	–	–	–	–	–	–	0.41
Turf	–	–	–	–	0.82	–	–	–	0.82
Unknown	–	–	–	–	7.55	–	–	–	7.55
Weed control	–	–	–	–	0.27	–	–	–	0.27
Total	1.51	3.30	0.96	0.14	92.45	0.41	0.82	0.41	100.00

Note

Sequenced DNA read counts were used as a proxy to semiquantify abundance. Samples are grouped into 13 categories, based on their type of impact in modified and natural systems (based on 97% similarity and e‐value ≥ 1–20 with NCIB database, BOLD sequences).

Abbreviations: Col, Coleoptera; Dip, Diptera; Hem, Hemiptera; Hym, Hymenoptera; Lep, Lepidoptera; Neu, Neuroptera; Ort, Orthoptera; Tri, Trichoptera.

Dietary prey species richness ranged from 24 to 228 per bat, with all richness estimators perhaps overestimating true richness (see Appendix S4). Lepidoptera (*n* = 673 species, 92.5% richness) were significantly more frequently consumed than any other arthropod order (*t* ≥ 1.202, *p*_(perm)_ ≤ .027 in all instances), with Diptera (24, 3.3%), Coleoptera (11, 1.5%), Hemiptera (7, 1.0%), Orthoptera (6, 0.8%), Trichoptera (3, 0.4%), Neuroptera (3, 0.4%), and Hymenoptera (1, 0.14%) also represented (Table 4). Noctuidae was the most frequently detected family (159 prey species, 22.8% richness). Frequently eaten species included the following: *Endotricha puncticostalis* (Lepidoptera: Pyralidae, *n* = 93% of samples); *Faveria tritalis* (Lepidoptera: Pyralidae, 88%); *Athetis tenuis* (Lepidoptera: Noctuidae, 84%); *Helicoverpa punctigera* (Lepidoptera: Noctuidae, 83%); *Tenebrio molitor* (Coleoptera: Tenebrionidae, 79%); *Achyra affinitalis* (Lepidoptera: Pyralidae, 76%); *Olbonoma* sp. (Lepidoptera: Oecophoridae, 74%); *Teleogryllus oceanicus* (Orthoptera: Gryllidae, 66%); and *Ectopatria horologa* (Lepidoptera: Noctuidae, 66%) (Table 3).

Male and female bats consumed a similar composition of prey items (Pseudo‐*F* = 1.394, *p*_(perm)_ = .172); however, males consumed a more diverse diet (Pseudo‐*F* = 1.600, *p*_(perm)_ = .030).

*Vespadelus vulturnus* (little forest bat, weight 3.9 g) and *N. geoffroyi* (lesser long‐eared bat, 8.2 g) consumed similar volumes of arthropods at the order level (Appendix S7), yet differences in prey species composition were near‐significant (*t* = 1.330, *p*_(perm)_ = .058). *V. vulturnus* notably consumed larger volumes of smaller‐sized prey items (tachinid flies, mosquitoes, gnats) than other bat species, while both *V. vulturnus* and *N. geoffroyi* consumed the most lygaeid bugs. *Chalinolobus morio* (chocolate wattled bat, 8.9 g) and *V. baverstocki* (inland forest bat, 4.6 g) consumed the most Noctuid moths in volume and richness*,* while *C. gouldii* (Gould's wattled bat, 13.8 g) consumed the greatest volume and richness of larger prey, such as crickets (Gryllidae). *Chalinolobus gouldii also* consumed a smaller range of prey items, with a greater relative abundance of Coleoptera than *V. vulturnus* or *N. geoffroyi* (Appendix S7). Significant differences in richness were detected between *C. gouldii* and *N. geoffroyi* using the species incidence matrix (Pseudo‐*F* = 1.339, *p*_(perm)_ = .023), indicating that rare prey items were responsible for the differences in diet.

**Figure 3:**
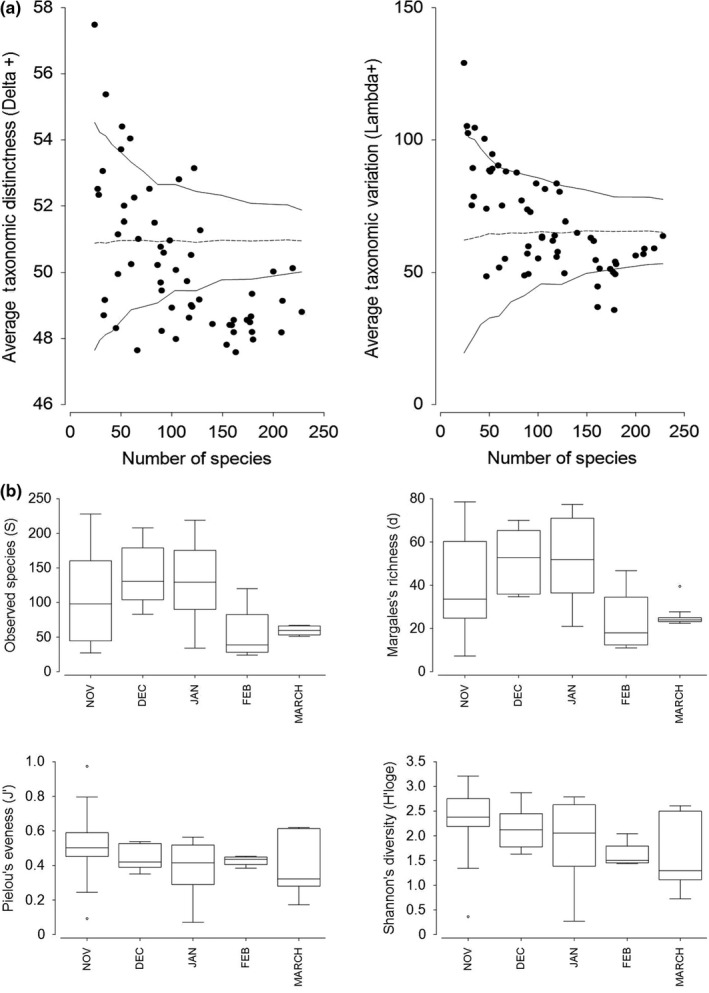
Taxonomic diversity and α diversity of bat diets. (a) Average taxonomic distinctness (left) and average taxonomic variation (right) against observed number of prey species in bat fecal samples, based on standardized, log (*X *+ 1)‐transformed data. Lines indicate median (dashed) and upper and lower 95% probability limits (continuous) for the simulated distribution intervals of Delta+ and Lambda+, created from the randomized list of 728 DNA detected prey species. (b) Box and whisker plots of α diversity measures by month over the cotton‐growing season. Based on log‐transformed prey species dataset with Euclidean distance matrix (Site 2 only), showing median (line in box), 25 and 75th percentiles, and 95% confidence intervals (whiskers)

Taxonomically, the analysis of AvTD showed that 43% of bat fecal samples had significantly lower AvTD than expected in relation to those species available in the habitat (*p* ≤ .05; Figure 3a). These bats thus exhibited strong dietary selectivity, consuming a narrower taxonomic diet in comparison with the prey available. All other bats had diets within the probability limits (or above) indicating a taxonomically diverse diet. However, 79% of samples fell within the 95% confidence limits of the VarTD funnel (Figure 3a), indicating similar variation in taxonomic distinctness of consumed prey species to those available. The estimated intercept for AvTD was 50.2 (±0.3 *SE*) and 68.8 (±2.5) for VarTD, meaning that, on average, prey species were related at family level for AvTD and at family/order level for VarTD (Table S6‐2).

### 3.5 Pest and beneficial arthropods in the diet of bats

Bats consumed significantly more agricultural pest arthropods than beneficial arthropods (all pests, *t* = 1.783, *p*_(perm)_ ≤ .001; cotton‐specific pests, *t* = 2.145, *p*_(perm)_ ≤ .001), with an average pest and beneficial volume of 65% ± 4.9% (*SE*) and 1% ± 0.6, respectively, per sample (Table 5). Of all 728 prey species, 94 species detected in bat fecal samples were pests of natural and modified systems, comprising 13% of prey species consumed (Table 6). In terms of relative abundance, these were mainly crop pests (45%, Table 4). Cotton‐specific pest arthropods comprised 19% of the dietary relative abundance and belonged to Lepidoptera (11%), Orthoptera (6%), and Hemiptera (1%) (Table 5). Of these, 19 cotton‐specific pest species from three arthropod orders were detected, including 11 species of Lepidoptera, three Hemiptera, and five Orthoptera, comprising 3% of species recorded (Table 5). Significant cotton pests such as *H. punctigera* (Australian bollworm) and *A. affinitalis* (cotton webspinner) were detected in at least 76% of bat fecal samples, with *T. oceanicus* (field crickets), *Helicoverpa armigera* (cotton bollworm), and *Crocidosema plebejana* (Tortricidae, cotton tipworm) detected in around 55% of bat fecal samples (Table 6). Other significant cotton pests including *Spodoptera exigua* (Noctuidae, lesser armyworm), *Nysius plebeius* (Lygaeidae, seed bug), *Creontiades dilutus* (Lygaeidae, green myrid), *Remaudiereana inornata* (Lygaeidae, seed bug), and *Mythimna oxygala* (Noctuidae, armyworm) were detected in at least 19% of bat fecal samples. The genus *Helicoverpa* was detected in 86% of bat fecal samples but at low average relative abundance (1.7%, range 0%–56%) per sample.

**Table 5:** Relative abundance (%) and richness (%) of pest and beneficial species by taxonomic order in bat fecal samples (based on 97% similarity and e‐value ≥ 1^–20^ with NCIB database, BOLD sequences)

Order	Modified and natural systems						Cotton‐specific arthropods					
	B	Bl	N	P	Pl	U	B	Bl	N	P	Pl	U
Relative abundance												
Coleoptera	0.00	0.03	0.09	13.88	–	–	–	0.03	13.03	–	0.94	–
Diptera	0.95	–	0.10	0.35	0.86	–	0.95	–	1.31	–	–	–
Hemiptera	–	–	0.21	1.68	0.00	–	–	–	0.21	1.68	0.00	–
Hymenoptera	–	–	0.00	–	–	–	–	–	0.00	–	–	–
Lepidoptera	0.04	–	21.08	42.69	9.85	1.51	0.00	–	62.75	10.65	0.37	1.41
Neuroptera	0.04	–	–	–	–	–	0.04	–	–	–	–	–
Orthoptera	–	–	–	6.57	–	–	–	–	0.02	6.55	–	–
Trichoptera	–	–	0.04	–	–	–	–	–	0.04	–	–	–
Total	1.04	0.03	21.53	65.18	10.72	1.51	1.00	0.03	77.36	18.88	1.32	1.41
Richness												
Coleoptera	0.14	0.27	0.41	0.69	–	–	–	0.27	0.96	–	0.27	–
Diptera	0.41	–	0.69	0.41	1.79	–	0.41	–	2.88	–	–	–
Hemiptera	–	–	0.41	0.41	0.14	–	–	–	0.41	0.41	0.14	–
Hymenoptera	–	–	0.14	–	–	–	–	–	0.14	–	–	–
Lepidoptera	0.41	–	68.54	10.58	5.36	7.55	0.14	–	82.28	1.51	1.37	7.14
Neuroptera	0.41	–	–	–	–	–	0.41	–	–	–	–	–
Orthoptera	–	–	–	0.82	–	–	–	–	0.14	0.69	–	–
Trichoptera	–	–	0.41	–	–	–	–	–	0.41	–	–	–
Total	1.37	0.27	70.60	12.91	7.28	7.55	0.96	0.27	87.23	2.61	1.79	7.14

Note

Sequenced DNA read counts were used as a proxy to semiquantify abundance.

Abbreviations: B, beneficial; Bl, beneficial likely; N, neutral (not pest or beneficial); P, pest; Pl, pest likely; U, unknown pest status.

**Table 6:** Arthropod pests (*n* = 94) of natural and modified systems detected in fecal samples (based on 97% similarity and e‐value ≥ 1–20 with NCIB database, BOLD sequences)

Scientific name	Order	Family	Type of impact to natural and modified systems	Known pest status: Cotton	Frequency of occurrence in bat fecal samples (%)	Average relative abundance (%)	Standard error (%)	Maximum relative abundance per sample (%)
*Tenebrio molitor*	Coleoptera	Tenebrionidae	Stored grain	Neutral	79.31	12.1	3.8	98.2
*Athetis tenuis*	Lepidoptera	Noctuidae	Crop	Neutral	84.48	4.8	1.8	78.1
*Teleogryllus oceanicus*	Orthoptera	Gryllidae	Crop	Pest	65.52	4.6	2.0	86.0
*Achyra affinitalis*	Lepidoptera	Pyralidae	Crop	Pest	75.86	4.5	2.1	83.4
*Faveria tritalis*	Lepidoptera	Pyralidae	Turf	Neutral	87.93	4.3	2.1	97.4
*Endotricha puncticostalis*	Lepidoptera	Pyralidae	Crop	Neutral	93.10	4.3	2.0	86.2
*Sceliodes cordalis*	Lepidoptera	Crambidae	Crop	Neutral	44.83	2.1	1.6	89.6
*Leucania stenographa*	Lepidoptera	Noctuidae	Crop	Neutral	44.83	1.7	1.5	85.8
*Tathorhynchus fallax*	Lepidoptera	Erebidae	Emergent	Neutral	62.07	1.6	0.9	42.0
*Helicoverpa punctigera*	Lepidoptera	Noctuidae	Crop	Pest	82.76	1.6	1.0	55.6
*Aproaerema isoscelixantha*	Lepidoptera	Gelechiidae	Emergent	Neutral	18.97	1.4	1.4	83.5
*Etiella behrii*	Lepidoptera	Pyralidae	Crop	Neutral	41.38	1.4	0.9	50.3
*Spodoptera exigua*	Lepidoptera	Noctuidae	Emergent	Pest	43.10	1.1	0.9	52.8
*Remaudiereana inornata*	Hemiptera	Lygaeidae	Emergent	Pest	18.97	0.7	0.7	39.3
*Procometis diplocentra*	Lepidoptera	Autostichidae	Forestry	Neutral	31.03	0.5	0.5	30.6
*Crocidosema plebejana*	Lepidoptera	Tortricidae	Crop	Pest	55.17	0.5	0.5	29.6
*Philobota chionoptera*	Lepidoptera	Oecophoridae	Crop	Neutral	48.28	0.5	0.3	13.6
*Merophyas divulsana*	Lepidoptera	Tortricidae	Crop	Neutral	31.03	0.4	0.3	18.4
*Sitona discoideus*	Coleoptera	Curculionidae	Crop	Pest likely	20.69	0.3	0.3	16.6
*Cryptochironomus* sp. *1 MEC‐2014*	Diptera	Chironominae	Crop	Neutral	17.24	0.3	0.2	13.8
*Isopteron* sp. *GJK‐2014*	Coleoptera	Tenebrionidae	Crop	Pest likely	15.52	0.2	0.2	10.8
*Creontiades dilutus*	Hemiptera	Miridae	Crop	Pest	18.97	0.2	0.2	8.9
*Helicoverpa armigera*	Lepidoptera	Noctuidae	Crop	Pest	58.62	0.2	0.1	6.3
*Xanthodes congenita*	Lepidoptera	Noctuidae	Emergent	Pest	6.90	0.1	0.1	7.2
*Nysius plebeius*	Hemiptera	Lygaeidae	Emergent	Pest	31.03	0.1	0.1	3.3
*Procometis hylonoma*	Lepidoptera	Autostichidae	Forestry	Neutral	27.59	0.1	0.1	4.8
*Earias huegeliana*	Lepidoptera	Nolidae	Crop	Pest	8.62	0.0	0.0	2.6
*Procometis* sp. *ANIC5*	Lepidoptera	Autostichidae	Forestry	Neutral	8.62	0.0	0.0	2.3
*Phaneroptera gracilis*	Orthoptera	Tettigoniidae	Crop	Neutral	5.17	0.0	0.0	1.1
*Mythimna* sp. *BOLD:AAY5769*	Lepidoptera	Noctuidae	Crop	Pest	18.97	0.0	0.0	1.1
*Parabagrotis cupidissima*	Lepidoptera	Noctuidae	Crop	Pest likely	37.93	0.0	0.0	0.6
*Creatonotos gangis*	Lepidoptera	Erebidae	Crop	Neutral	20.69	0.0	0.0	1.1
*Philobota orescoa*	Lepidoptera	Oecophoridae	Crop	Neutral	18.97	0.0	0.0	0.8
*Paranisitra longipes voucher MNHN‐EO‐ENSIF3157*	Orthoptera	Gryllidae	Crop	Pest	6.90	0.0	0.0	0.5
*Teleogryllus infernalis*	Orthoptera	Gryllidae	Crop	Pest	24.14	0.0	0.0	0.3
*Scopula rubraria*	Lepidoptera	Geometridae	Crop	Neutral	5.17	0.0	0.0	0.4
*Scopula optivata*	Lepidoptera	Geometridae	Crop	Neutral	8.62	0.0	0.0	0.4
*Hippotion celerio*	Lepidoptera	Sphingidae	Orchard	Neutral	18.97	0.0	0.0	0.4
*Spodoptera ochrea*	Lepidoptera	Noctuidae	Crop	Pest likely	15.52	0.0	0.0	0.4
*Swammerdamia caesiella*	Lepidoptera	Yponomeutidae	Orchard	Neutral	31.03	0.0	0.0	0.1
*Epyaxa hyperythra*	Lepidoptera	Geometridae	Crop	Neutral	15.52	0.0	0.0	0.2
*Typhaea stercorea*	Coleoptera	Mycetophagidae	Stored grain	Neutral	8.62	0.0	0.0	0.1
*Feraxinia nyei*	Lepidoptera	Noctuidae	Crop	Neutral	5.17	0.0	0.0	0.2
*Xestia oblata*	Lepidoptera	Noctuidae	Emergent	Neutral	8.62	0.0	0.0	0.1
*Mythimna oxygala*	Lepidoptera	Noctuidae	Crop	Pest	18.97	0.0	0.0	0.2
*Diarsia rubifera*	Lepidoptera	Noctuidae	Emergent	Neutral	37.93	0.0	0.0	0.0
*Xestia speciosa*	Lepidoptera	Noctuidae	Emergent	Neutral	17.24	0.0	0.0	0.1
*Lobesia vanillana*	Lepidoptera	Tortricidae	Orchard	Neutral	15.52	0.0	0.0	0.1
*Xanthorhoe decoloraria*	Lepidoptera	Geometridae	Crop	Neutral	29.31	0.0	0.0	0.0
*Scopula* sp. *ANIC3*	Lepidoptera	Geometridae	Crop	Neutral	18.97	0.0	0.0	0.1
*Velarifictorus beybienkoi*	Orthoptera	Gryllidae	Crop	Pest	20.69	0.0	0.0	0.0
*Philobota* sp. *ANIC98*	Lepidoptera	Oecophoridae	Crop	Neutral	17.24	0.0	0.0	0.0
*Acleris chalybeana*	Lepidoptera	Tortricidae	Orchard	Neutral	25.86	0.0	0.0	0.0
*Endotricha flammealis*	Lepidoptera	Pyralidae	Crop	Pest likely	22.41	0.0	0.0	0.0
*Leucania anteroclara*	Lepidoptera	Noctuidae	Emergent	Neutral	8.62	0.0	0.0	0.1
*Scopula decorata*	Lepidoptera	Geometridae	Crop	Neutral	8.62	0.0	0.0	0.1
*Philobota* sp. *ANIC31*	Lepidoptera	Oecophoridae	Crop	Neutral	15.52	0.0	0.0	0.0
*Mythimna separata*	Lepidoptera	Noctuidae	Crop	Pest	8.62	0.0	0.0	0.0
*Philobota* sp. *ANIC146*	Lepidoptera	Oecophoridae	Crop	Neutral	20.69	0.0	0.0	0.0
*Peribatodes rhomboidaria*	Lepidoptera	Geometridae	Crop	Neutral	31.03	0.0	0.0	0.0
*Etiella scitivittalis*	Lepidoptera	Pyralidae	Crop	Neutral	13.79	0.0	0.0	0.0
*Lobesia nr. transtrifera 11ANIC‐11910*	Lepidoptera	Tortricidae	Orchard	Neutral	10.34	0.0	0.0	0.0
*Abagrotis variata*	Lepidoptera	Noctuidae	Orchard	Neutral	24.14	0.0	0.0	0.0
*Sesamia nonagrioides*	Lepidoptera	Noctuidae	Crop	Neutral	24.14	0.0	0.0	0.0
*Acrobasis* sp. *ANIC1*	Lepidoptera	Pyralidae	Orchard	Neutral	17.24	0.0	0.0	0.0
*Philobota* sp. *ANIC188*	Lepidoptera	Oecophoridae	Crop	Neutral	12.07	0.0	0.0	0.0
*Philobota protorthra*	Lepidoptera	Oecophoridae	Crop	Neutral	10.34	0.0	0.0	0.0
*Cryptolestes ferrugineus*	Coleoptera	Laemophloeidae	Stored grain	Neutral	1.72	0.0	0.0	0.0
*Tiracola plagiata*	Lepidoptera	Noctuidae	Emergent	Neutral	8.62	0.0	0.0	0.0
*Macrobathra leucopeda*	Lepidoptera	Cosmopterigidae	Disease vector	Neutral	13.79	0.0	0.0	0.0
*Mythimna* sp. *BOLD:AAQ0235*	Lepidoptera	Noctuidae	Crop	Pest	17.24	0.0	0.0	0.0
*Odontodes aleuca*	Lepidoptera	Noctuidae	Emergent	Neutral	22.41	0.0	0.0	0.0
*Ochlerotatus sticticus*	Diptera	Culicidae	Disease vector	Neutral	5.17	0.0	0.0	0.0
*Philobota cirrhopepla*	Lepidoptera	Oecophoridae	Crop	Neutral	1.72	0.0	0.0	0.0
*Stenoma* sp. *Janzen20*	Lepidoptera	Depressariidae	Orchard	Neutral	6.90	0.0	0.0	0.0
*Xestia smithii*	Lepidoptera	Noctuidae	Emergent	Neutral	22.41	0.0	0.0	0.0
*Acleris curvalana*	Lepidoptera	Tortricidae	Orchard	Neutral	5.17	0.0	0.0	0.0
*Spodoptera frugiperda*	Lepidoptera	Noctuidae	Crop	Pest likely	17.24	0.0	0.0	0.0
*Blastobasis tarda*	Lepidoptera	Blastobasidae	Orchard	Neutral	12.07	0.0	0.0	0.0
*Etiella zinckenella*	Lepidoptera	Pyralidae	Crop	Neutral	6.90	0.0	0.0	0.0
*Senometopia nr. cinerea Shima01*	Diptera	Tachinidae	Forestry	Neutral	1.72	0.0	0.0	0.0
*Teleogryllus emma*	Orthoptera	Gryllidae	Crop	Pest	8.62	0.0	0.0	0.0
*Etiella hobsoni*	Lepidoptera	Pyralidae	Crop	Neutral	12.07	0.0	0.0	0.0
*Philobota* sp. *ANIC191*	Lepidoptera	Oecophoridae	Crop	Neutral	5.17	0.0	0.0	0.0
*Philobota zalias*	Lepidoptera	Oecophoridae	Crop	Neutral	1.72	0.0	0.0	0.0
*Apamea apamiformis*	Lepidoptera	Noctuidae	Crop	Neutral	13.79	0.0	0.0	0.0
*Hellula hydralis*	Lepidoptera	Pyralidae	Crop	Neutral	6.90	0.0	0.0	0.0
*Hulstia undulatella*	Lepidoptera	Pyralidae	Crop	Neutral	3.45	0.0	0.0	0.0
*Etiella walsinghamella*	Lepidoptera	Pyralidae	Crop	Neutral	1.72	0.0	0.0	0.0
*Philobota argotoxa*	Lepidoptera	Oecophoridae	Crop	Neutral	8.62	0.0	0.0	0.0
*Macrochilo orciferalis*	Lepidoptera	Erebidae	Orchard	Neutral	5.17	0.0	0.0	0.0
*Philobota* sp. *ANIC156*	Lepidoptera	Oecophoridae	Crop	Neutral	6.90	0.0	0.0	0.0
*Blastobasis catapealla*	Lepidoptera	Blastobasidae	Orchard	Neutral	1.72	0.0	0.0	0.0
*Hydraecia ximena*	Lepidoptera	Noctuidae	Emergent	Neutral	3.45	0.0	0.0	0.0

Note

DNA read counts were used as a proxy to semiquantify relative abundance of a prey item.

### 3.6 Temporal variation in bat diet

Dietary composition based on relative abundance of prey species differed significantly between early–mid season (November–January) and late season (March) (Pseudo‐*F* = 1.90, *p*_(perm)_ ≤ .001). The composition of arthropods collected in light traps was significantly different over the growing season (Pseudo‐*F* = 3.977, *p*_(perm)_ ≤ .001), with March significantly different to other months. The increased relative abundance of Coleoptera and decrease in Lepidoptera in March was the primary driver of the difference. Coleoptera was the most abundant order in light trap collections during all months (> 40% by volume per month) except January, when Lepidoptera were most abundant (51%; Figure 2c). However, the mean relative abundance of Lepidoptera in bat fecal samples over the growing season (> 58%) did not differ significantly between months (Pseudo‐*F* = 0.555, *p*_(perm)_ = .710; Figure 2b), indicating that bats consumed consistent proportions of Lepidoptera over the cotton‐growing season, despite a pronounced temporal shift in the composition of available arthropod prey orders in cotton crops. The RELATE analysis confirmed no relationship between the proportions of arthropods (at order level) eaten by insectivorous bats and those collected in light traps over the growing season (ρ = – 0.081, *p* = .545).

A significant difference in total dietary α diversity (*d*) was detected between the middle and end of the cotton‐growing season (December–January vs. February–March) (*t* ≥ 2.707, *p*_(perm)_ ≤ .039 for all times). Alpha diversity generally declined over the cotton season, with the lowest diversity in March (corresponding with cotton defoliation; Figure 3b). Dietary β diversity did not vary significantly among months (*F* = 2.842, *p*_(perm)_ = .256).

## 4 DISCUSSION

Our study demonstrates the complementary role that qualitative and semiquantitative interpretation of metabarcoding sequence reads can provide in uncovering the nuances of prey items consumed by insectivorous bats. Our results support a growing body of global evidence illustrating the significant role that insectivorous bats play in arthropod pest control. Our results also emphasize the service, rather than disservice, bats provide to agriculture, consuming a diet comprised of around 1% relative abundance and richness of beneficial insects (predators, parasitoids, pollinators). Thus, based on pest versus beneficial insect consumption alone, the benefits of bat‐mediated insect suppression in crops outweigh any disservice. Importantly, 19 cotton‐specific pest species, in three arthropod orders, were detected. The most abundant arthropods in the diet of insectivorous bats were pests of summer crops, suggesting that bats were sourcing arthropods from cotton and other summer‐grown crops. Furthermore, insectivorous bats consumed few unique species and many species only once, supporting evidence that bats exploit preferred locally abundant taxa (such as large pest moth population influxes) in agriculture while simultaneously consuming a wide selection of available prey (Krauel et al., 2018).

Dietary composition (average relative abundance and richness) was dominated by Lepidoptera and did not change significantly over the growing season, irrespective of fluctuations in moth abundance in the landscape (Figure 2). Importantly, bats continued to consume high proportions of lepidopterans during March, even though light trap data suggested a decrease in availability of lepidopterans in cotton crops at this time and an increase in Coleoptera (Figure 2). These results suggest that bats selectively predated lepidopterans and likely reflect an adjustment in the habitat where moths were sourced, since lepidopteran abundance declined in cotton crops late in the season. The significant cotton pest moth genus, *Helicoverpa,* did not dominate bat diets in this study. This was likely a function of the low abundance of *Helicoverpa* in the study region rather than selective foraging. Contrary to Federico et al. (2008), we suggest that bats relying on specific invertebrate influxes (i.e., *Helicoverpa* sp. and other Lepidoptera) to meet energetic requirements are not significantly affected by Bt‐cotton in Australian agroecosystems due to their ability to adjust foraging behavior to hunt moths elsewhere in the landscape.

Bats also frequently consumed soft‐bodied flies, but these do not contribute greatly to overall diet in terms of volume, as found in similar studies (Gonsalves, Bicknell, Law, Webb, & Monamy, 2013a; Rydell, McNeill, & Eklöf, 2002; Wetzler & Boyles, 2017). Small prey items such as mosquitoes and gnats were most common in the diet of *V. vulturnus* (the smallest species of bat), while larger prey items (such as crickets) were most common in the diet of *C. gouldii* (a larger aerial‐hawking bat) and *N. geoffroyi* (a gleaning bat). The ability of these bats to detect insect prey is constrained by echolocation call structure and explains the variation in prey choice (Waters, Rydell, & Jones, 1995). Smaller bats tend to use high‐frequency‐modulated “soft” echolocation and are restricted to detecting smaller prey items, typically dipterans and moths 5–10 mm in length (Gonsalves, Bicknell, et al., 2013a; Møhl, 1988; Robert & Brigham, 1991). However, several studies on larger aerial‐hawking bats that echolocate using low‐frequency and long‐duration calls show that they consume a range of prey sizes (Waters et al., 1995). In addition, bats use echolocation counterstrategies (such as reducing the amplitude or shifting frequencies) to exploit difficult‐to‐catch prey that are typically tympanate “hearing” insects (Goerlitz, Hofstede, Zeale, Jones, & Holderied, 2010; Miller & Surlykke, 2001). Noctuid moths formed a major component of the diet of insectivorous bats in this study, despite the ability of Noctuidae to hear, avoid, respond, and block the echolocation calls of approaching bats (Jacobs & Bastian, 2017). This finding supports existing studies where Noctuidae have been detected in high abundance in bat diets globally (Bohm, Wells, & Kalko, 2011; Dodd, Chapman, Harwood, Lacki, & Rieske, 2012; Rolfe, Kurta, & Clemans, 2014; Wickramasinghe, Harris, Jones, & Vaughan Jennings, 2004). This further demonstrates how echolocation avoidance by tympanate insects can be overcome by bats in order to exploit large food resources (Goerlitz et al., 2010; Miller & Surlykke, 2001; Surlykke & Kalko, 2008). For example, *B. barbastellus* (an aerial‐hawking bat) consumes mainly tympanate moths using low‐intensity echolocation, thus detecting moths at a closer distance allowing them less time to respond or escape capture (Goerlitz et al., 2010). Other strategies to counter moth hearing include shifting echolocation frequency out of the audible range of prey (Fenton & Fullard, 1981), broadening the echolocation beam when in the final hunting phase (Jakobsen, Olsen, & Surlykke, 2015) or changing hunting strategy (Van De Sijpe & Holsbeek, 2007). The counterstrategies employed by bats against tympanate insects may explain how *C. gouldii* appears inconspicuous to hearing moths and crickets, consuming large amounts (volume and richness) as indicated by their diet in this study. *N. geoffroyi* consumes crickets through a combination of allotonic (frequency‐mismatched) calls, together with high‐frequency short‐wave echolocation >70 kHz (less detectable frequencies) (Fullard, Ratcliffe, & Guignion, 2005). Furthermore, crickets are unable to exhibit echolocation evasion responses while on the ground, that is, not in flight (Fullard et al., 2005), and are thus susceptible to gleaning bats such as *N. geoffroyi*.

The diversity of echolocation in bats and thus detection of insect prey provide strong support for the maintenance of bat functional diversity in agricultural areas. Increased predator functional diversity improves natural pest control (Barbaro et al., 2017; Greenop, Woodcock, Wilby, Cook, & Pywell, 2018) and is important for the suppression of a range of pest insect species in crops. This is because bats exert different pressures on different insects, mediated by echolocation constraints and thus the detection and capture of prey (Waters et al., 1995). The magnitude of bat insect pest suppression changes during the night, with bats’ timing roost emergence to coincide with access to preferred prey (Swift, Racey, & Avery, 1985), but is dependent on predation risk, light intensity, and life stage (Duvergé, Jones, Rydell, & Ransome, 2000; Rydell, Entwistle, & Racey, 1996). Farmers wishing to benefit from the insect pest control service provided by bats in the cotton‐growing landscape can incorporate bat‐mediated insect suppression into existing IPM strategies by managing a diversity of noncrop habitat and roosting sites to support different bat species foraging over crops.

The general pattern of decline in dietary α diversity over the cotton‐growing season reflected the change in arthropod community composition (increased Coleoptera and reduced Lepidoptera) as the crop matured (Figure 2). The changing structure of the arthropod community over the cotton‐growing season is likely to drive temporal and spatial α and β diversity in bat diets, as bats have less opportunity to hunt preferred lepidopteran prey. This temporal flexibility may provide agricultural economic benefits as bats could potentially target large pest populations as they irrupt. More importantly, large pest outbreaks may also benefit bats in agricultural zones (Monck‐Whipp, Martin, Francis, & Fahrig, 2018) by increasing access to insect prey and foraging habitat during the summer reproductive season. Arthropod population studies in concert with insectivorous bat dietary analysis and spatial foraging information would assist in understanding the spatiotemporal complexity of these interactions in Bt‐cotton landscapes.

Despite the broad range of arthropods consumed by insectivorous bats, our results suggest considerable overlap in shared prey resources in terms of the most abundant prey taxa consumed irrespective of species and sex. This indicates that resource partitioning was low. While there was some evidence of bat species differences driving fine‐scale dietary changes (likely a function of foraging style), the effect on diet composition was not clear, due to limited sample size. Shared prey resources are uncommon in insectivorous bats as differences in species morphology and echolocation behavior dictate habitat use and diet (Aldridge & Rautenbach, 1987; Reside & Lumsden, 2001). Nevertheless, this study suggests that various species of insectivorous bat can coexist in agroecosystems when abundant preferred prey taxa are available. Further studies are required to determine whether fine‐scale resource partitioning occurs between insectivorous bats (Adams & Thibault, 2006) utilizing the prey resources in cotton landscapes. Despite low‐resource portioning, our results indicate that male bats consume a more diverse diet than females, confirming that females show more selective feeding when faced with abundant food resources, such as crop pests (Anthony & Kunz, 1977; Czenze et al., 2018).

### 4.1 Metabarcoding and taxonomic identity limitations

The limitations of molecular methods for dietary studies are well recognized (Krehenwinkel et al., 2017; Pompanon et al., 2012), and interpreting estimates of taxon abundance from sequence reads remains a challenge. The number of relative reads (OTUs) may not perfectly reflect dietary prey composition as a result of species differences in the amount of DNA per unit of mass or volume; digestibility and hence DNA degradation during digestion; amplification success; and recovery bias. However, it can be used to offer a semiquantitative view of diet composition and variation (Deagle et al., 2018).

Taxonomic resolution in metabarcoding‐type studies of diets is limited by the choice of primer. Broad‐spectrum primers may amplify nontarget species including amplicons from the predator, gut parasites, and symbionts (Deagle et al., 2007; Pompanon et al., 2012). However, primer mismatches may lead to the overrepresentation of some prey taxa or preferential amplification of other taxa and thus provide significant differences in read abundances across taxa (Deagle et al., 2007; Krehenwinkel et al., 2017). The limitations of the Zeale et al. (2011) primers used in this study have been tested in various dietary studies targeting the barcoding CO1 region, and the primers have produced reliable arthropod species lists (Alberdi et al., 2018). Amplification bias can be mitigated by the use of degenerate COI primers that can provide a more reliable qualitative and quantitative recovery of DNA reads and thus reveal species diversity (Elbrecht & Leese, 2017; Krehenwinkel et al., 2017). Nevertheless, the Zeale primers show no significant arthropod amplification bias (Zeale et al., 2011). Despite this, it is currently disputed whether abundance estimates can be derived from metabarcoding due to taxon‐specific PCR amplification biases (Krehenwinkel et al., 2017). One solution is to use taxon‐specific correction factors, which allow species abundances to be predicted from sequencing data (Thomas et al., 2016). However, with a generalist predator consuming a diverse diet, characterizing the taxonomic composition of a large community of prey would not be feasible. Several recent studies have shown a strong correlation between input DNA and recovered read counts for most arthropod taxa (Giner et al., 2016; Krehenwinkel et al., 2017). Thus, no correction factor was applied to our data and we used the number of reads assigned to a species on the NCBI database as a proxy for the semiquantification of prey items in terms of relative abundance (Deagle et al., 2018). While our estimates of relative abundance may differ from the true biomass proportions of taxa in the diets of the bats, we are confident in the major patterns of variation observed.

## 5 CONCLUSION

Australian insectivorous bats have a diverse diet in transgenic cotton landscapes (made up of a few key species and many unique species) dominated by Lepidoptera in a major cotton production zone in inland eastern Australia. This suggests that insectivorous bats are capable of selective foraging on preferred taxa (moths). Selective foraging behavior that adjusts to the available prey over the growing season benefits both bats and agriculture, as bats have access to a wider prey resource and can target pest moth population outbreaks. Our results show that bat diets were dominated by pest arthropods of economic importance. This provides evidence for growers to integrate insectivorous bats into pest management programs, provided that bats and their habitat are conserved. Vegetation management strategies that incorporate insectivorous bat habitats and bat‐friendly farm management practices are vital in maintaining and maximizing these important pest control services in intensive farming regions.

## CONFLICT OF INTEREST

None declared.

## AUTHOR CONTRIBUTIONS

HK, RS, RR, and NR designed the research and experiments. HK planned the fieldwork, collected and analyzed the data, and drafted the manuscript. All authors contributed substantially to the analytical approach and manuscript revisions and gave final approval for publication.

## Data Availability

The OTU and MegaBLAST data that support the findings of this study are available for download from Dryad, https://doi.org/10.5061/dryad.jsxksn05f.
