# Supplementary material for: Insectivorous bats selectively source moths and eat mostly pest insects on dryland and irrigated cotton farms
Source: Ecol Evol. 2019 Dec 12;10(1):371–88. doi: 10.1002/ece3.5901 (PMC6972826; doi:10.1002/ece3.5901)
Supplement: Supplementary file 1 [file ECE3-10-371-s001.docx]

# SUPPORTING INFORMATION

# Appendix S1 – Additional method and analysis details

## Methods and materials

## Study sites

Site 1 (3147 ha) was located on black and grey self-mulching soils on the Gwydir alluvial plains (Mitchell, 2002). Remnant vegetation in the vicinity was predominantly classified as forest, floodplain woodland and semi-arid grassland (Keith, 2004). Native vegetation adjacent to cropping fields included natural grasslands largely dominated by *Austrostipa aristiglumis* (plains grass), with woodland fragments dominated by *Casuarina cristata* (belah), *Allocasuarina luehmannii* (buloke), *Eucalyptus populnea* (poplar box), *Acacia stenophylla* (black wattle), *Geijera parviflora* (wilga) and *Callitris glaucophylla* (white cypress pine) in addition to other tree species and occasional *Acacia pendula* (myall). Millie Creek (an ephemeral creek) located on the southern property boundary was lined with *Acacia salicina* (cooba) and *A. stenophylla*. Site 2 (1645 ha) was located on the cracking clays and alluvial soils of the Liverpool Plains (Mitchell, 2002). Remnant vegetation was predominantly western slopes grassy woodland and inland riverine forests dominated by *E. camaldulensis* (river red gum) associated with the Namoi River (Keith, 2004). Native vegetation adjacent to crops was a mix of planted and remnant vegetation dominated by *E. populnea, E. melliodora* (yellow box), *G. parviflora* and *A. pendula* with open grasslands dominated by *A. aristiglumis*, *Chloris truncata, Bothriochloa sp., Panicum spp*., and *Dichanthium sericeum* (Qld blue grass) on black vertosols.

## Data analysis

### Dietary richness and relative abundance

The use of both relative abundance and richness of prey items overcomes limitations with each measurement method. Relative abundance can underestimate the volume of prey species not easily digested, whilst richness can overestimate the importance of small prey items (Klare et al., 2011). A parallel analysis of dietary diversity was undertaken of these two abundance datasets. Log(x+1) transformed abundance data and presence–absence metrics with a Bray–Curtis similarity matrix (for multivariate tests) or Euclidean distance similarity matrix (for univariate tests) were used. This allowed emphasis to be placed on both relative abundance and species composition (weighting rare vs common species equally).

Alpha (*α*) diversity of samples from both datasets was measured using five common diversity indices: (1) total species (*S*); (2) Shannon’s diversity (*H′log = – Σ pi* log *(pi)*), where *pi* was the proportion of the total count arising from the *i* th species; (3) Simpson’s diversity *(1–λ′ = 1 – {Σi Ni (Ni–1) } / {N(N–1)})*, where *Ni* was the number of individuals of species *i*; (4) Margalef’s richness (*d = (S – 1) / log N*) and (5) Pielou’s evenness *J = H′ / log S*): (Appendix S8). A PERMANOVA using a Euclidean distance matrix was constructed on the diversity measures to compare temporal and spatial *α* diversity (between farms over the cotton growing season) using both datasets. Variability in composition (*β* diversity) was measured using PERMDISP on a Bray–Curtis similarity matrix (presence/absence data), which is equivalent to the Sørensen index (Anderson 2006; Anderson et al. 2008).

### Abundance of landscape prey

PERMANOVA (Bray–Curtis similarity matrix) and PERMDISP were performed on the standardised light trap dataset. The RELATE routine (a non-parametric Mantel-type statistic) with a Pearson’s correlation coefficient (ρ) was used (999 permutations) to assess the relationship between prey available and prey eaten by bats (Clarke & Gorley, 2015). The light trap dataset and taxonomically assigned prey species dataset were condensed to relative abundance of arthropod orders by month and a Bray–Curtis similarity matrix constructed for matrix comparison.

### Limitations of reference database

Taxonomic assignment was constrained by the limitations of the reference database. The COI coding region has the most widely available sequence region in public reference libraries. Yet, it is estimated that over 100,000 Australian insect species have not yet been described (Yeates, 2016) and as such, will not occur in the NCBI database. Although Australian cotton pest arthropods are well-known, several have not had their DNA sequenced (including *Campylomma liebknechti* apple dimpling bug, *Nysius vinitor* Rutherglen bug and *Deraeocoris signatus* brown smudge bug). Thus, adding these species to the database and developing species-specific primers would assist in reliably identifying the impact of insectivorous bats on major Australian cotton pests both qualitatively and semi-quantitatively. A custom reference database of food items was not feasibly possible in this study given the potential breadth of food items (Pompanon et al., 2012)

### Diversity indices and taxonomic distinctness

The weighting of Linnaean tree-step lengths (species, genus, family, order, class and phylum) was based on equal step lengths (*ω* = 16·67) and guided by taxon richness of the species dataset. All factors (month, site, sex, species) were plotted on the AvTD and VarTD array to explore data patterns. AvTD was defined as: Δ+ = [ ΣΣ *i* ***<*** *j* ω*i j* ] / [ *S* (*S* − 1) / 2 ] (Clarke & Warwick, 1998; Clarke et al., 2014), where ΣΣ was the double summation ranges over all pairs *i* and *j* of these species (i < j), *ω i j* was the weight given to the length of the path which joins the species *i* and *j* in the taxonomy and *S* was the observed number of species in the sample. VarTD was defined as Λ+ = [ ΣΣ *i* ***<*** *j* (ω *i j* – Δ+) 2 ] / [ *S* (*S* − 1) / 2 ], where the variance of the taxonomic distances was measured (ω*ij*) over all pairs *i* and *j* (i < j) of these species from their mean value Δ+ (Clarke et al., 2014).

**References**

Clarke, K. R. & Warwick, R. M. 1998. A taxonomic distinctness index and its statistical properties. *J Appl Ecol,* 35**,** 523–531.

Clarke, K. R., Gorley, R. N., Somerfield, P. J. & Warwick, R. M. 2014. *Change in marine communities: an approach to statistical analysis and interpretation,*, PRIMER-E: Plymouth.

Clarke, K. R. & Gorley, R. N. 2015. *PRIMER v7: User Manual/Tutorial,* Plymouth, UK: PRIMER-E.

Klare, U., Kamler, J. F. & Macdonald, D. W. 2011. A comparison and critique of different scat-analysis methods for determining carnivore diet. *Mammal Review,* 41**,** 294-312.

Pompanon, F., Deagle, B. E., Symondson, W. O., Brown, D. S., Jarman, S. N. & Taberlet, P. 2012. Who is eating what: diet assessment using next generation sequencing. *Mol Ecol,* 21**,** 1931–50.

Yeates, D. 2016. Why so many Australian species are yet to be named. *The Conversation.*

# Appendix S2 – Pest and beneficial arthropod definitions

The pest or beneficial status of prey species was determined through a literature review. Beneficial taxa were defined as those that provide a beneficial service to agricultural, human or natural systems by predating or parasitising a pest taxon or providing a pollination service. Pest taxa were defined as those species which are considered detrimental to agricultural, human or natural systems or have a negative economic impact (i.e. reduced yield, cause destruction). Prey items were further categorised as pest or beneficial arthropod specific to cotton farms in Australia according to Deutscher *et al.* (2004) and Williams *et al.* (2011).

**Table S2-1: Pests, disease vectors and beneficial arthropods in modified and natural systems. Literature sources: Alford, 2016; Cooke et al., 2009; Bailey, 2007; Hill, 2010; Horne & Page, 2008; NSW DPI, 2017; QLD DAF, 2017; Rees & Rangsi, 2004.**

|  | **Category** | **Agricultural/Forestry/Horticultural inclusions in this category** |
| --- | --- | --- |
| **Pest** | Crop | Cotton, corn, rice, peanut, beets, mung beans, lucerne, sweet potato, brassica, cereals, coffee, pastures, |
|  | Turf | Grasses, turf, |
|  | Forestry | Plantations (eucalypt, pine), woodland reserves |
|  | Orchard | Grapes, fruit, citrus, tomato, capsicum, eggplant, avocado |
|  | Disease vector | Spread disease |
|  | Invasive | Listed invasive species |
|  | Emergent | Future pests. Based on Paini et al. (2010) |
|  | Timber | Destructive (e.g. termites) |
|  | Stored grain | Grain, stored products |
| **Beneficial** | Weed control | Introduced species to control invasive herbaceous weeds |
|  | Pollination | Known pollinator in agricultural systems |
|  | Parasitic | Parasitic life stage |
|  | Predator | Predator |

**References**

Alford, D. V. 2016. *Pests of Fruit Crops: A Colour Handbook*, Second Edition, CRC Press.

Bailey, P. 2007. *Insects of field crops and pastures in Australia*, Collingwood: Vic, Australia, CSIRO Pub.

Cooke, T., Persley, D. & House, S. 2009. Diseases of Fruit Crops in Australia, Collingwood: Vic, Australia, CSIRO Pub.

Deutscher, S., Wilson, L. & Mensah, R. 2004. *Integrated Pest Management Guidelines for Cotton Production Systems in Australia*, Paragon Printers Australasian, The Australian Cotton Cooperative Research Centre.

Hill, D. S. 2010. *Pests of Crops in Warmer Climates and Their Control*, Springer Netherlands.

Horne, P. A. & Page, J. 2008. *Integrated Pest Management for Crops and Pastures*, Landlinks.

NSW DPI. 2017. *Insect pests*. Department of Primary Industries. Available: http://www.dpi.nsw.gov.au/biosecurity/insect-pests [Accessed June 2017].

Paini, D., Worner, S. & Cook, D. 2010. *Early warning of pre-emergent emergency plant pest threats*. Cooperative Research Centre for National Plant Biosecurity.

Qld DAF. 2017. Department of Agriculture and Fisheries Managing insect pests in field crops: A-Z insect pest list. Available: https://www.daf.qld.gov.au/plants/field-crops-and-pastures/broadacre-field-crops/integrated-pest-management/a-z-insect-pest-list [Accessed June 2017].

Rees, D. P. & Rangsi, T. V. 2004. *Insects of Stored Products*, Collingwood: Vic, Australia, CSIRO Pub.

Williams, S., Wilson, L. & Vogel, S. 2011. *Pests & beneficials in Australian cotton landscapes*, The Development and Delivery Team (Cotton Catchment Communities CRC), Cotton Grower Services and the IHD Group.

# Appendix S3 – Analysis of OTU dataset

OTU richness ranged from 54 to 544 per sample, with Lepidoptera (n = 1623), Coleoptera (384) and Diptera (289) the most frequently recorded orders (Table S6-1). Dietary composition based on OTU abundance was significantly different between location (Pseudo-F = 2.64, P_(perm)_ ≤ 0.01) and month (Pseudo-F = 1.43, P_(perm)_ ≤ 0.01) between early and late in the cotton growing season. OTU dietary composition was significantly different between *Nytophilus geoffroyi* and *Chalinolobus gouldii* but did not vary with sex. No significant difference in *α* diversity (J, H’log or d) was detected between months, farms, bat species or sex. No significant difference in *β* diversity of OTUs was detected between month (Pseudo-F = 2.8, P_(perm)_ = 3.0), farm (Pseudo-F = 3.3, P(perm) = 0.18), bat species (t ≥ 0.25, P_(perm)_ ≥ 0.45 in all cases) or sex (t = 0.82, P_(perm)_ = 0.47). There was weak evidence showing a lower mean *α* diversity in bat diet at the end of the growing season (March) than the beginning (December). Similar results were obtained using the species-assigned dataset, suggesting that no resolution was lost between datasets and that OTU identity to species level did not bias the findings.

**Table S3-1:** Relative abundance and richness of Operational Taxonomic Units (OTUs) and assigned taxonomic species (n = 728) grouped into arthropod orders (based on 97% and e-value ≥ 1-20 similarity with NCIB database, BOLD sequences). Orders with < 2 OTUs have been excluded from the table.

| **Phylum**  **Class**  Order | **OTU read proportion (%)** | **OTU richness** | **Species richness** | **Species richness (%)** | **Species relative abundance (n=728) (%)** |
| --- | --- | --- | --- | --- | --- |
| **Arthropoda** | **99.78** | **2754** | **728** | **100.00** | **100.00** |
| ***Insecta*** | ***99.71*** | ***2752*** | ***728*** | ***100.00*** | ***100.00*** |
| Araneae | 0.25 | 7 | - | - | - |
| Blattodea | 1.78 | 49 | - | - | - |
| Coleoptera | 13.95 | 385 | 11 | 1.51 | 14.00 |
| Decapoda | 0.11 | 3 | - | - | - |
| Diptera | 10.47 | 289 | 24 | 3.30 | 2.26 |
| Hemiptera | 3.77 | 104 | 7 | 0.96 | 1.89 |
| Hymenoptera | 0.25 | 7 | 1 | 0.14 | 0.00 |
| Lepidoptera | 58.8 | 1623 | 673 | 92.45 | 75.17 |
| Neuroptera | 0.25 | 7 | 3 | 0.41 | 0.04 |
| Orthoptera | 5.98 | 165 | 6 | 0.82 | 6.57 |
| Trichoptera | 0.87 | 24 | 3 | 0.41 | 0.04 |
| Unknown | 2.97 | 82 | - | - | - |
| **Total (including excluded OTUs)** | **100** | **2760** | **728** | **100** | **100** |

# Appendix S4 – Species accumulation curves and K-dominance plots


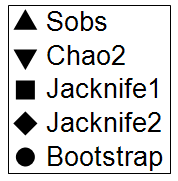
**
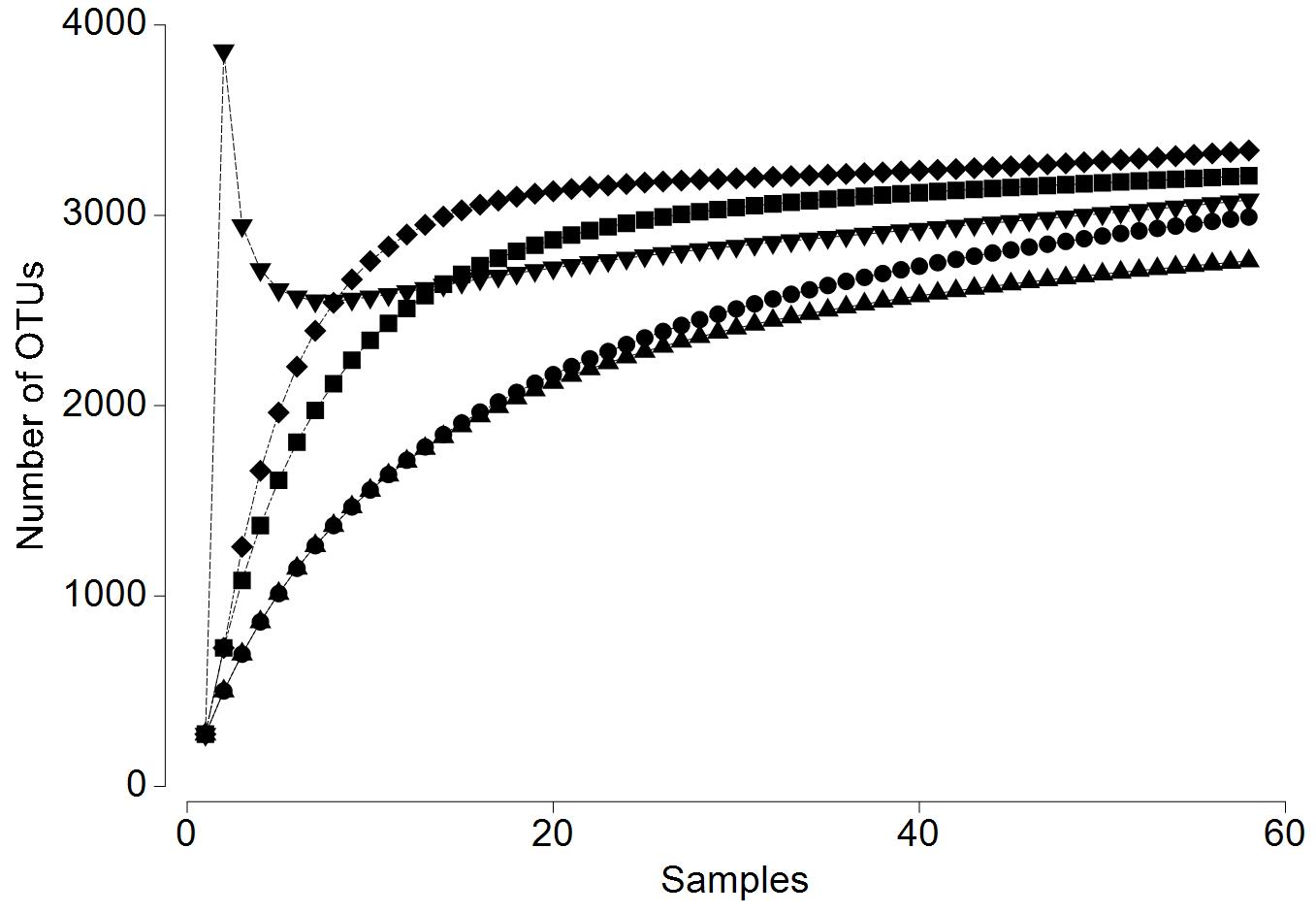
**

a)


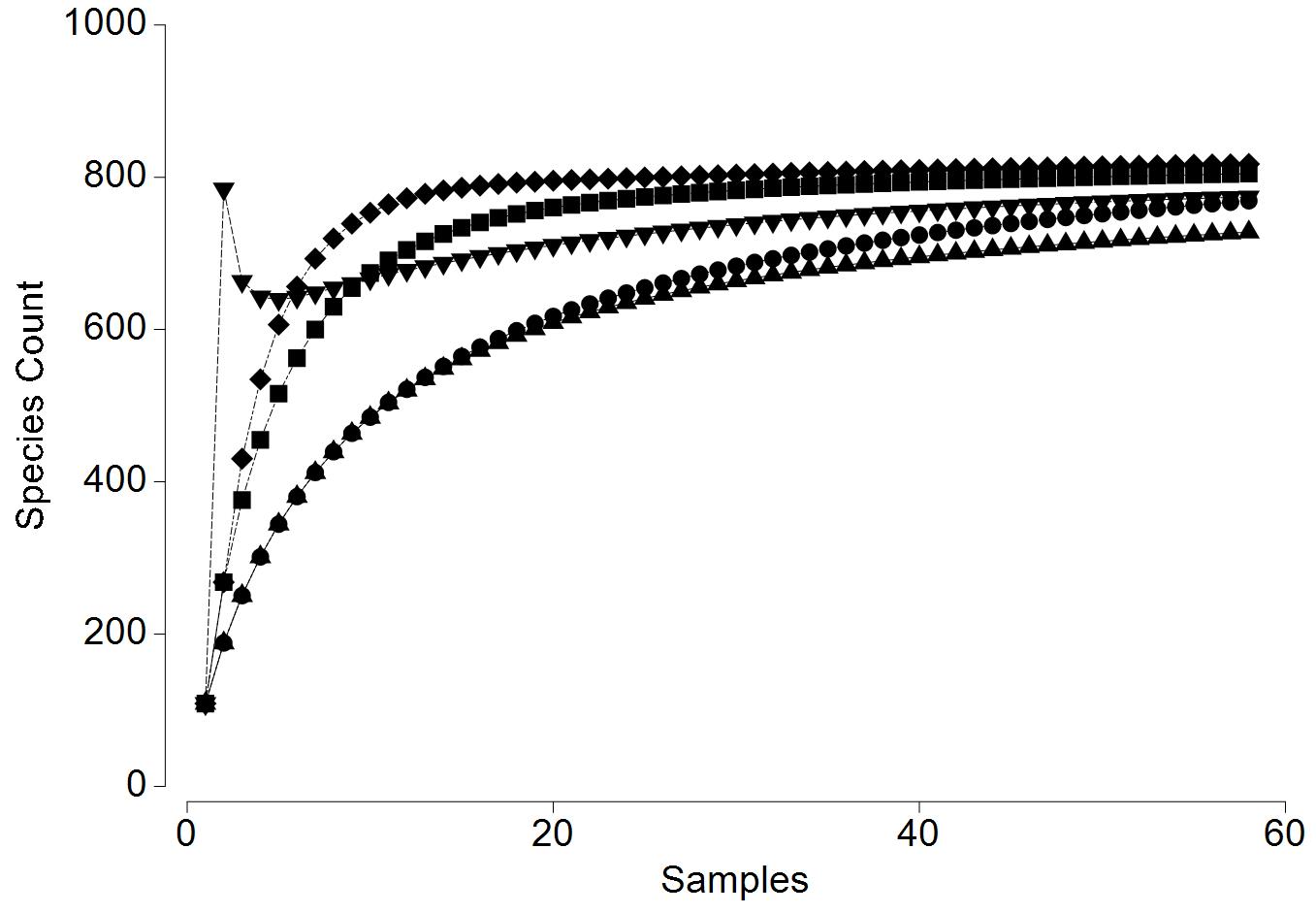


b)

**
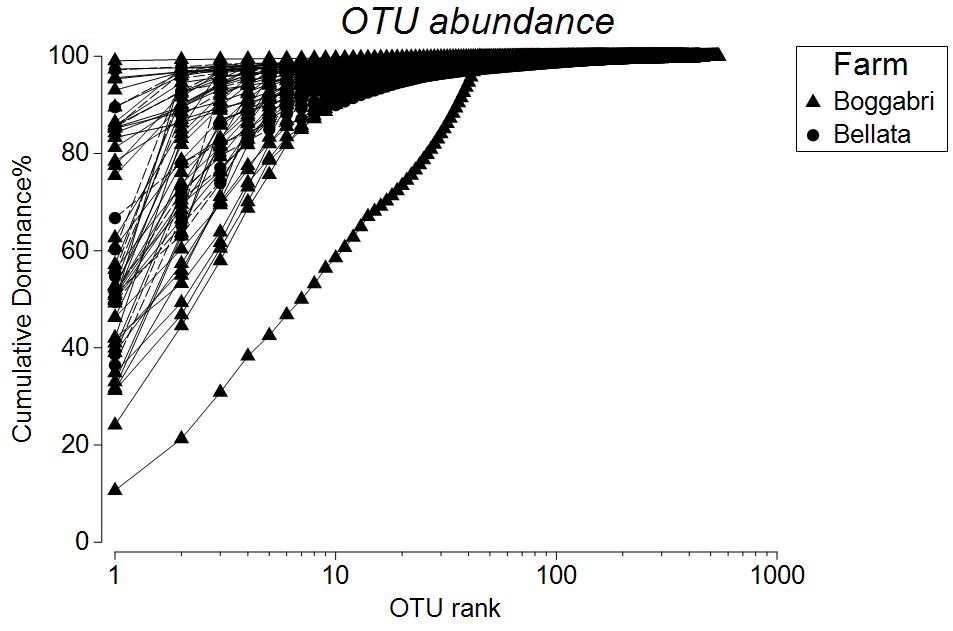
**

c)

**
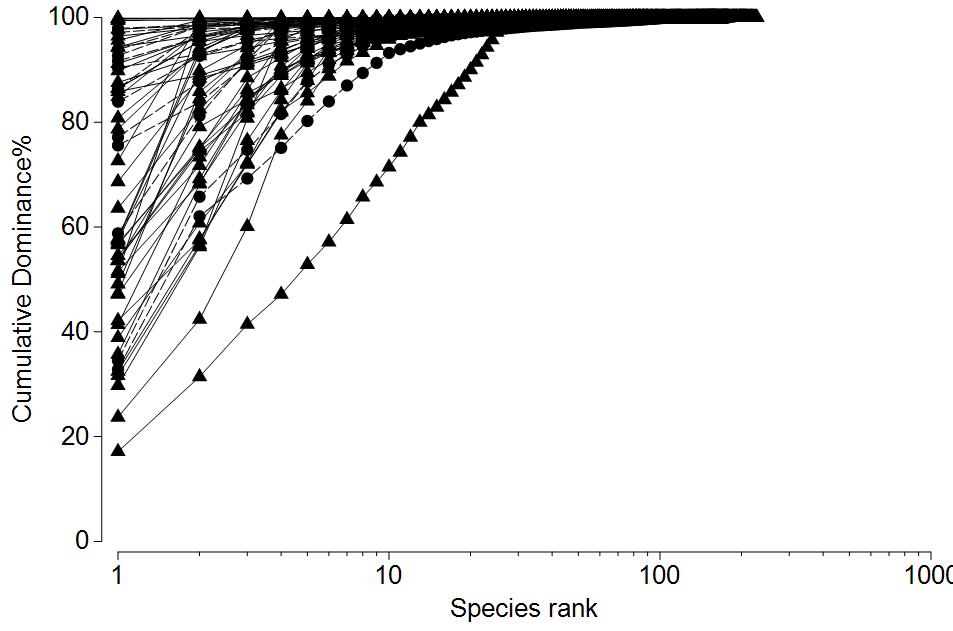
**

d)

**Figure S4-1: a)** OTU accumulation curve, **b)** species accumulation curve based on individual bat samples. The *y*-axis shows the increasing total number of different species observed (S), based on 97% similarity with NCIB database, as samples are successively pooled, **c)** K-Dominance curves for bat faecal samples based on OTUs, and **d)** taxonomically assigned species. Each curve is based on prey species (or OTU) abundances in a single sample. By 19 samples, >2082 OTUs had been covered by all estimators, indicating that sampling had reached 75% richness and suggesting a sufficient sampling effort of prey species. The number of duplicate OTUs was high (Chao2, Jacknife1 and Jacknife2). The Bootstrap estimator overestimated the number of observed species (Sobs).

# Appendix S5 – Spatial variations in bat diet

The spatial comparison between two farms over different years is considered an appropriate comparison, given that the cotton insect community is well known and changes with the phenology of the crop. A significant difference in prey composition between farm locations (Pseudo-F = 3.888, P_(perm)_ ≤ 0.001) was detected indicating that the type and volume of prey consumed at each farm significantly contributed to the variation in bat diet. At the order level, the suite of most abundant arthropods varied spatially. After Lepidoptera, Coleoptera (17.2%), Orthoptera (11.0%) and Diptera (3.3%) dominated dietary prey at Site 2, whereas Orthoptera (7.3%), Diptera (4.8%) and Coleoptera (2.0%) dominated diet at Site 1. Furthermore, microbats consumed a less diverse, more similar prey species assemblage at Site 2 than Site 1. At Site 1 the average similarity between prey species was 17.3%, and prey primarily consisted of *Endotricha puncticostalis*, *Cyana sp. BOLD:AAN5000* (Lepidoptera: Erebidae), *Sturmiopsis parasitica* (Diptera: Tachinidae) and *Olethreutes fasciatana* (Lepidoptera: Tortricidae). At Site 2, the average similarity between prey species was 23.8%, with prey primarily consisting of *Athetis tenuis*, *Helicoverpa punctigera* and *Etiella behrii* (Lepidoptera: Pyralidae). The spatial variation in dietary composition shows that although many of the same prey taxa were present in faecal samples, they were not consumed in the same proportions. No significant difference in α diversity (*d, h’log* or *J*) or β diversity (F = 6.676, P_(perm)_ = 0.065) was detected between farms over the two years (December 2015 and 2016).

# Appendix S6 – Diversity calculations

**Table S6-1: Diversity indices as calculated by e-PRIMER. Based on the standardised log (X+1) transformed microbat prey species dataset**

|  | **S** | **N** | **d** | **J'** | **H'(loge)** | **1-Lambda'** |
| --- | --- | --- | --- | --- | --- | --- |
| 1 | 27 | 36.00 | 7.26 | 0.97 | 3.21 | 0.98 |
| 2 | 98 | 18.02 | 33.55 | 0.52 | 2.38 | 0.92 |
| 3 | 28 | 19.42 | 9.10 | 0.73 | 2.42 | 0.93 |
| 4 | 115 | 14.96 | 42.13 | 0.45 | 2.16 | 0.89 |
| 5 | 50 | 4.89 | 30.89 | 0.09 | 0.36 | 0.14 |
| 6 | 179 | 20.66 | 58.78 | 0.54 | 2.82 | 0.94 |
| 7 | 86 | 17.06 | 29.96 | 0.49 | 2.20 | 0.91 |
| 8 | 228 | 17.98 | 78.57 | 0.50 | 2.73 | 0.93 |
| 9 | 154 | 10.65 | 64.68 | 0.44 | 2.23 | 0.85 |
| 10 | 157 | 10.90 | 65.31 | 0.39 | 1.97 | 0.82 |
| 11 | 208 | 19.22 | 70.03 | 0.54 | 2.87 | 0.94 |
| 12 | 83 | 10.68 | 34.63 | 0.40 | 1.78 | 0.82 |
| 13 | 104 | 12.19 | 41.19 | 0.35 | 1.63 | 0.82 |
| 14 | 179 | 15.89 | 64.36 | 0.44 | 2.27 | 0.89 |
| 15 | 104 | 17.65 | 35.88 | 0.53 | 2.45 | 0.92 |
| 16 | 177 | 16.20 | 63.19 | 0.46 | 2.36 | 0.91 |
| 17 | 47 | 4.86 | 29.08 | 0.09 | 0.34 | 0.13 |
| 18 | 47 | 4.78 | 29.40 | 0.07 | 0.27 | 0.09 |
| 19 | 89 | 13.16 | 34.15 | 0.41 | 1.86 | 0.85 |
| 20 | 209 | 15.89 | 75.20 | 0.49 | 2.61 | 0.92 |
| 21 | 180 | 10.97 | 74.73 | 0.35 | 1.84 | 0.80 |
| 22 | 90 | 6.64 | 47.02 | 0.21 | 0.95 | 0.55 |
| 23 | 174 | 9.35 | 77.39 | 0.41 | 2.14 | 0.80 |
| 24 | 117 | 21.90 | 37.58 | 0.56 | 2.65 | 0.94 |
| 25 | 34 | 4.83 | 20.96 | 0.09 | 0.32 | 0.11 |
| 26 | 200 | 20.66 | 65.72 | 0.53 | 2.79 | 0.94 |
| 27 | 159 | 18.62 | 54.03 | 0.53 | 2.69 | 0.94 |
| 28 | 92 | 13.20 | 35.26 | 0.55 | 2.50 | 0.91 |
| 29 | 219 | 22.26 | 70.26 | 0.51 | 2.75 | 0.95 |
| 30 | 161 | 9.31 | 71.73 | 0.39 | 1.97 | 0.79 |
| 31 | 119 | 19.19 | 39.94 | 0.56 | 2.69 | 0.94 |
| 32 | 140 | 6.56 | 73.91 | 0.31 | 1.56 | 0.59 |
| 33 | 163 | 14.48 | 60.62 | 0.45 | 2.29 | 0.90 |
| 34 | 100 | 7.32 | 49.75 | 0.26 | 1.22 | 0.64 |
| 35 | 90 | 9.41 | 39.70 | 0.40 | 1.80 | 0.78 |
| 36 | 32 | 9.66 | 13.67 | 0.44 | 1.54 | 0.79 |
| 37 | 45 | 7.25 | 22.22 | 0.38 | 1.46 | 0.67 |
| 38 | 120 | 12.77 | 46.72 | 0.43 | 2.04 | 0.86 |
| 39 | 24 | 8.10 | 11.00 | 0.45 | 1.44 | 0.73 |
| 40 | 66 | 5.18 | 39.50 | 0.17 | 0.72 | 0.26 |
| 41 | 51 | 8.64 | 23.19 | 0.28 | 1.11 | 0.66 |
| 42 | 67 | 19.04 | 22.40 | 0.62 | 2.61 | 0.94 |
| 43 | 60 | 10.60 | 24.99 | 0.36 | 1.47 | 0.78 |
| 44 | 53 | 8.34 | 24.52 | 0.28 | 1.11 | 0.65 |
| 45 | 59 | 12.04 | 23.31 | 0.61 | 2.50 | 0.90 |
| 46 | 33 | 9.05 | 14.53 | 0.50 | 1.75 | 0.76 |
| 47 | 161 | 10.26 | 68.72 | 0.40 | 2.04 | 0.84 |
| 48 | 53 | 13.38 | 20.05 | 0.50 | 1.99 | 0.86 |
| 49 | 35 | 12.17 | 13.61 | 0.67 | 2.39 | 0.89 |
| 50 | 178 | 14.29 | 66.56 | 0.52 | 2.67 | 0.91 |
| 51 | 63 | 9.29 | 27.81 | 0.46 | 1.89 | 0.80 |
| 52 | 128 | 21.16 | 41.61 | 0.56 | 2.71 | 0.95 |
| 53 | 107 | 25.10 | 32.89 | 0.67 | 3.15 | 0.97 |
| 54 | 78 | 7.95 | 37.14 | 0.34 | 1.50 | 0.70 |
| 55 | 122 | 14.72 | 44.99 | 0.53 | 2.53 | 0.91 |
| 56 | 119 | 6.37 | 63.71 | 0.27 | 1.31 | 0.55 |
| 57 | 127 | 7.74 | 61.56 | 0.31 | 1.52 | 0.69 |
| 58 | 89 | 6.99 | 45.26 | 0.39 | 1.75 | 0.65 |

**Table S6-2: Weightings for AvTD and VarTD as calculated by e-PRIMER. Based on the standardised log (X+1) transformed prey species dataset**

| Taxon | Branch | Weight |
| --- | --- | --- |
| Scientific name | 1 | 16.667 |
| Genus | 1 | 33.333 |
| Family | 1 | 50 |
| Order | 1 | 66.667 |
| Class | 1 | 83.333 |
| Phylum | 1 | 100 |

**Table S6-3: AvTD and VarTD as calculated by e-PRIMER. Based on the standardised log (X+1) transformed prey species dataset.**

|  |  | Av TD | | Var TD | |
| --- | --- | --- | --- | --- | --- |
| Sample | S | Δ+ Value | Δ+ Sig | Λ+ Value | Λ+ Sig |
| 1 | 27 | 52.52 | 35.00 | 105.25 | 4.60 |
| 2 | 98 | 50.97 | 96.90 | 83.51 | 7.60 |
| 3 | 28 | 52.34 | 37.00 | 102.56 | 4.80 |
| 4 | 115 | 49.74 | 12.00 | 62.01 | 79.30 |
| 5 | 50 | 53.71 | 3.60 | 88.47 | 12.60 |
| 6 | 179 | 49.35 | 0.80 | 49.36 | 3.80 |
| 7 | 86 | 50.23 | 45.60 | 48.89 | 17.80 |
| 8 | 228 | 48.81 | 0.20 | 63.81 | 76.30 |
| 9 | 154 | 47.81 | 0.20 | 63.12 | 78.90 |
| 10 | 157 | 48.41 | 0.20 | 61.95 | 68.30 |
| 11 | 208 | 48.19 | 0.20 | 56.96 | 21.00 |
| 12 | 83 | 51.51 | 52.70 | 77.07 | 26.00 |
| 13 | 104 | 47.99 | 0.20 | 63.11 | 83.90 |
| 14 | 179 | 48.20 | 0.20 | 54.11 | 13.80 |
| 15 | 104 | 50.08 | 27.00 | 63.63 | 85.90 |
| 16 | 177 | 48.49 | 0.20 | 50.08 | 5.00 |
| 17 | 47 | 51.16 | 85.50 | 73.95 | 59.50 |
| 18 | 47 | 49.95 | 44.80 | 48.56 | 33.20 |
| 19 | 89 | 50.78 | 84.30 | 73.66 | 42.40 |
| 20 | 209 | 49.14 | 0.20 | 59.01 | 31.40 |
| 21 | 180 | 47.97 | 0.20 | 53.16 | 9.40 |
| 22 | 90 | 48.23 | 0.20 | 59.92 | 63.90 |
| 23 | 174 | 48.56 | 0.20 | 51.35 | 6.60 |
| 24 | 117 | 48.63 | 0.20 | 63.99 | 96.50 |
| 25 | 34 | 49.17 | 25.00 | 78.53 | 45.40 |
| 26 | 200 | 50.03 | 8.20 | 56.41 | 16.40 |
| 27 | 159 | 48.41 | 0.20 | 54.70 | 16.20 |
| 28 | 92 | 50.60 | 71.50 | 72.70 | 46.20 |
| 29 | 219 | 50.13 | 9.60 | 59.13 | 31.20 |
| 30 | 161 | 48.56 | 0.20 | 44.71 | 2.00 |
| 31 | 119 | 50.53 | 57.70 | 83.51 | 6.40 |
| 32 | 140 | 48.44 | 0.20 | 65.00 | 99.10 |
| 33 | 163 | 47.59 | 0.20 | 51.48 | 11.20 |
| 34 | 100 | 48.94 | 1.60 | 55.32 | 34.00 |
| 35 | 90 | 49.45 | 7.00 | 49.43 | 16.60 |
| 36 | 32 | 53.06 | 19.40 | 75.22 | 54.30 |
| 37 | 45 | 48.32 | 2.40 | 100.42 | 1.60 |
| 38 | 120 | 48.96 | 1.00 | 57.83 | 45.00 |
| 39 | 24 | 57.49 | 0.60 | 129.12 | 0.60 |
| 40 | 66 | 47.65 | 0.20 | 55.19 | 45.80 |
| 41 | 51 | 54.41 | 0.60 | 88.00 | 12.20 |
| 42 | 67 | 51.02 | 95.10 | 88.04 | 7.60 |
| 43 | 60 | 50.25 | 54.10 | 51.88 | 37.60 |
| 44 | 53 | 52.01 | 41.20 | 89.10 | 12.00 |
| 45 | 59 | 54.04 | 1.00 | 90.32 | 5.80 |
| 46 | 33 | 48.71 | 14.20 | 89.34 | 16.40 |
| 47 | 161 | 48.19 | 0.20 | 36.94 | 0.20 |
| 48 | 53 | 51.54 | 54.70 | 94.60 | 4.00 |
| 49 | 35 | 55.38 | 0.40 | 104.60 | 1.60 |
| 50 | 178 | 48.67 | 0.20 | 35.81 | 0.20 |
| 51 | 63 | 52.25 | 25.60 | 75.14 | 45.20 |
| 52 | 128 | 51.28 | 61.50 | 69.24 | 64.30 |
| 53 | 107 | 52.81 | 2.60 | 81.42 | 9.40 |
| 54 | 78 | 52.52 | 10.40 | 87.63 | 4.40 |
| 55 | 122 | 53.14 | 0.80 | 80.37 | 10.60 |
| 56 | 119 | 49.01 | 0.60 | 55.92 | 37.20 |
| 57 | 127 | 49.18 | 1.00 | 49.71 | 9.60 |
| 58 | 89 | 49.70 | 16.80 | 57.15 | 46.80 |
| mean |  | 50.2 |  | 68.8 |  |
| Standard deviation |  | 2.1 |  | 19.0 |  |
| Standard error |  | 0.3 |  | 2.5 |  |

# Appendix S7 – Dietary richness and relative abundance of three insectivorous bat species

**Figure S7**–**1: a)** **Box and whisker plots of richness of prey items in the diet of the three most common insectivorous bat species captured. Box shows median (line in box), 25^th^ and 75^th^ percentiles and 95% confidence intervals (whiskers) b) Average relative abundance of arthropods (based on 97% similarity and e-value ≥ 1^–20^ with NCIB database, BOLD sequences) grouped to order level. Sample sizes: *Vespadelus vulturnus*, n = 19; *Nyctophilus geoffroyi*, n = 14; *Chalinolobus gouldii,* n = 6.**

*N geoffroyi*

*C. gouldi*

*V. vulturnus*


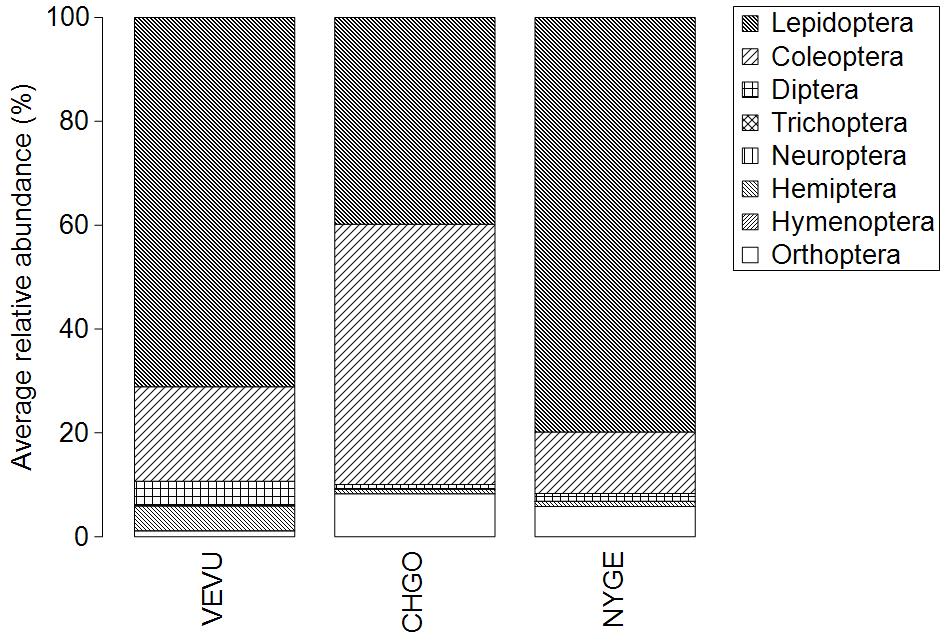


b)


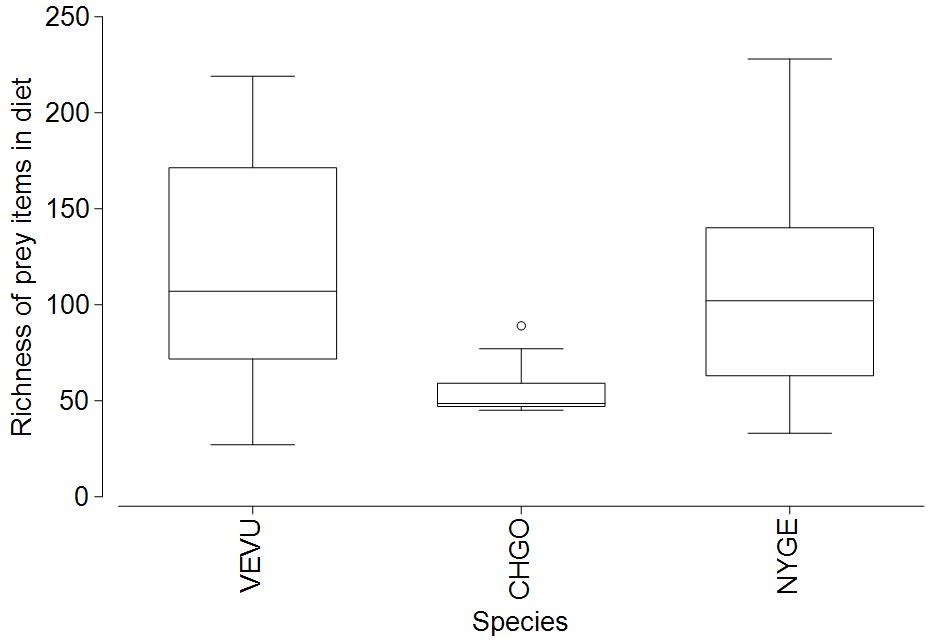


a)

*V. vulturnus*

*C. gouldii*

*N. geoffroyi*
